# Lower Respiratory Microbiome Dysbiosis Is Associated With Poor Prognosis in Acute Severe Lower Respiratory Tract Infection

**DOI:** 10.1002/mco2.70907

**Published:** 2026-08-09

**Authors:** Minghua Zhan, Hongbin Chen, Ziyao Li, Si Liu, Binghuai Lu, Zhang Wang, Hui Wang

**Affiliations:** ^1^ Department of Clinical Laboratory Peking University People's Hospital Beijing China; ^2^ Hebei Key Laboratory of Pathogenic Mechanisms and Diagnosis & Treatment Technologies for Lung Microbiome, Department of Clinical Laboratory First Affiliated Hospital of Hebei North University Zhangjiakou Hebei China; ^3^ Laboratory of Clinical Microbiology and Infectious Diseases, Department of Pulmonary and Critical Care Medicine, Center of Respiratory Medicine, National Clinical Research Center for Respiratory Diseases, National Center for Respiratory Medicine, Institute of Respiratory Medicine China–Japan Friendship Hospital Beijing China; ^4^ Chinese Academy of Medical Sciences Beijing China; ^5^ Institute of Ecological Sciences School of Life Sciences South China Normal University Guangzhou Guangdong China

**Keywords:** acute severe lower respiratory tract infections, immune responses, lower respiratory tract microbiome, mNGS

## Abstract

Acute severe lower respiratory tract infections (asLRTIs) pose a significant clinical challenge, especially in critically ill patients, but the role of the lower respiratory tract microbiome (LRTM) remains unclear. This study aimed to characterize LRTM composition and host immune factors to identify prognostic features of clinical outcomes. The study included 53 asLRTI patients and 35 controls. Metagenomics, metabolomics, proteomics, and RNA sequencing were conducted, while analysis of similarities (ANOSIM) and Cox regression were performed for statistics. Clinical data, including pneumonia severity scores, were collected on BALF sampling, with a 100‐day follow‐up. LRTM samples were grouped into five clusters (C1–C5). Cluster C5 resembled controls, while others showed significantly lower diversity. LRTM composition correlated with prognosis, with higher pathogenic bacteria abundance linked to poorer outcomes. Cluster C3 was associated with poor prognosis and reduced survival. Metabolite analysis revealed elevated α‐ketoisocaproic acid in asLRTIs and higher 10‐nitrolinoleate in poor‐prognosis patients. Immune responses varied across clusters, with distinct gene and cytokine expression patterns. Cluster C1, associated with *Acinetobacter baumannii*, exhibited heightened IL17 pathway activation. LRTM composition in asLRTIs is linked to clinical outcomes, with no single gradient of difference but distinct community states characterized by varying pathogens, metabolites, and immune responses.

## Graphical abstract

Acute severe lower respiratory tract infection (asLRTI) present lower alpha‐diversity in lower respiratory tract microbiome (LRTM). Five clusters (C1–C5) were grouped, and cluster C3 was strongly associated with poor prognosis and reduced survival. Cluster C1, associated with *Acinetobacter baumannii*, exhibited heightened IL17 pathway activation.

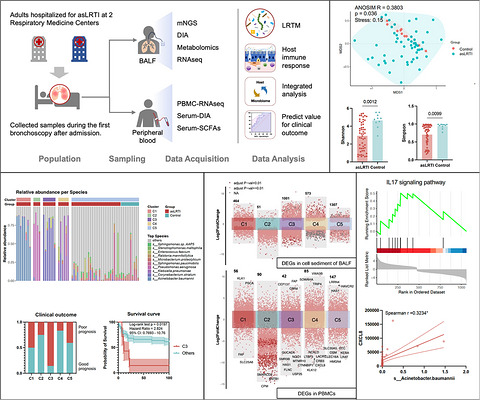

## 1 Introduction

Acute severe lower respiratory tract infection (asLRTI) is a common but serious respiratory infectious disease characterized by dyspnea and hypoxemia caused by inflammation of the alveoli and bronchi and is ranked sixth among the causes of death in all ages worldwide [1]. LRTIs are characterized as the combination of high microbial burden, low microbial diversity, and host inflammatory response from present lung microbiome studies [2, 3, 4, 5]. Dysbiosis of the lower respiratory tract microbiome (LRTM) is more serious and negatively related to disease severity specifically in critically ill patients [6].

As shown in previous studies, changes in the abundance and composition of LRTM are closely related to host immune responses, such as aggravated alveolar inflammation and progressive lung injury [7, 8, 9]. Therefore, we hypothesized that both LRTM and host immune responses would influence the prognosis of patients with asLRTIs. Montassier et al. identified different microbial species in acute respiratory distress syndrome (ARDS) and hospital‐acquired pneumonia (HAP), and screened the microbial community characteristics of patients with early indications for successful extubation [10]. Carney et al. predicted the risk of bacterial superinfection in individuals with COVID‐19 using machine learning based on blood transcriptome data [11].

To the best of our knowledge, few studies have conducted combined research on LRTM and host immune responses in the lungs during asLRTI. In this study, we collected bronchoalveolar lavage fluid (BALF) from patients with asLRTIs admitted for the first time. Using metagenomic next‐generation sequencing (mNGS) and RNA sequencing (RNA‐seq), we screened the differentially abundant microbiota and differentially expressed genes (DEGs) in asLRTIs and elucidated the pathological mechanisms that could be related to clinical outcomes.

## 2 Results

### 2.1 asLRTIs Present Unique Landscape‐Filled Interactions Between Microbial Communities and Host Immune System

**FIGURE 1:**
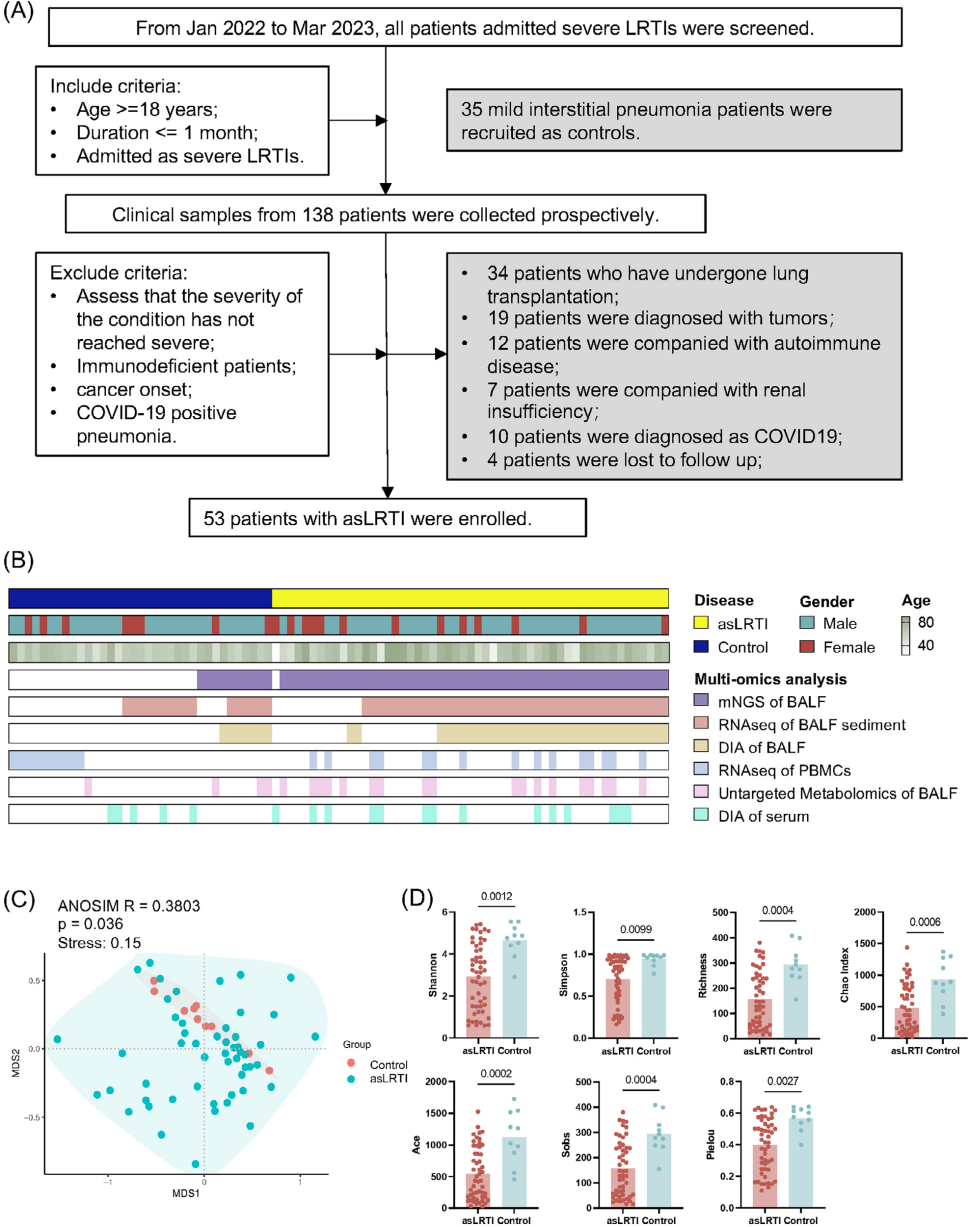
Decreased diversity of LRTM in asLRTIs. (A) Flow diagram of the study population. (B) Baseline demographic and distribution of patients who underwent multi‐omics. (C) LRTM in asLRTI was significantly differentiated from controls by NMDS based on Bray–Curtis dissimilarity (*p* = 0.036). Statistics method for NMDS plot is ANOSIM. ANOSIM *R* > 0 means the distance within the group is less than the distance between groups, indicating that the grouping is valid. (D) Alpha diversity indexes were significantly decreased in asLRTI (*p* < 0.05). *p* value was calculated by *t*‐test. *p* < 0.05 was defined as significant. Abbreviations: ANOSIM, analysis of similarities; asLRTIs, acute severe lower respiratory tract infections; BALF, bronchoalveolar lavage fluid; COVID19, coronavirus disease 2019; DIA, data‐independent acquisition; LRTM, lower respiratory tract microbiome; mNGS, metagenomic next‐generation sequencing; NMDS, nonmetric multidimensional scaling; PBMCs, peripheral blood mononuclear cells.

To explore the landscape of lower respiratory tract in asLRTIs, 53 patients admitted as asLRTIs and 35 patients with mild non‐COVID‐19 interstitial pneumonia were enrolled in this study (Figure 1A). Baseline demographic and distribution of patients who underwent multi‐omics are shown in Figure 1B and Table S1. First, LRTM were assessed by mNGS on 62 BALF from 52 asLRTIs and 10 controls. As shown in Table 1, significantly higher pneumonia severity index (PSI) and more BALF culture positive percentage were observed in asLRTIs (*p* < 0.0001, *p* = 0.0016). Of the patients with asLRTIs, 86.5% received mechanical ventilation, whereas 53.8% underwent tracheotomy (Table 1). Inflammation‐related markers, including white blood cells count, neutrophils, C‐reactive protein (CRP), and procalcitonin (PCT), were notably elevated in asLRTIs, accompanied by accelerated deterioration of physical function, characterized by decreased hemoglobin and elevated blood urea nitrogen (BUN) (Table 1). Noticeably, LRTM in asLRTIs was significantly different from that in the controls (analysis of similarities [ANOSIM] *R* = 0.3803, *p* = 0.036) (Figure 1C). Alpha diversity indices of LRTM in asLRTI were significantly decreased, including the Shannon, Simpson, Richness, Chao, Ace, Sobs, and Pielou indices (*p* < 0.05) (Figure 1D). Expansion of pathogenic bacteria was the main feature of LRTM in asLRTIs, including *Acinetobacter baumannii*, *Corynebacterium striatum*, *Pseudomonas aeruginosa*, and *Klebsiella pneumoniae* (Figure S1A).

**TABLE 1:** Clinical characteristics of patients undergoing Metagenomic Next‐Generation Sequencing (mNGS) in this study.

	asLRTI	Controls	*p* value^*^
**No**.	52	10	
**Gender (Male, %)**	41 (78.8)	8 (80.0)	0.9999
**Age**	73 (64, 79)	69 (61, 74)	0.1379
**From onset to admission (days)**	15 (6, 38)	30 (5, 46)	0.5670
**PSI**	110 (102, 120)	79 (68, 96)	**<0.0001**
Mechanical ventilation (%)	45 (86.5)	1 (10.0)	**<0.0001**
**Tracheotomy (%)**	28 (53.8)	0 (0.0)	**0.0013**
**Complications**			
Hypertension	25 (48.1)	4 (40.0)	0.7379
Diabetes	21 (40.4)	3 (30.0)	0.7272
Coronary heart disease	12 (23.1)	4 (40.0)	0.2661
Interstitial lung disease	6 (11.5)	3 (30.0)	0.1509
Hyperlipidemia	6 (11.5)	2 (20.0)	0.6043
Pulmonary tuberculosis	5 (9.6)	1 (10.0)	0.9999
COPD	2 (3.8)	0 (0.0)	0.9999
Bronchial asthma	1 (1.9)	1 (10.0)	0.2988
Bronchiectasis	1 (1.9)	0 (0.0)	0.9999
**Smoke**	21 (40.4)	5 (50.0)	0.7292
**BALF culture positive**	34 (65.4)	1 (10.0)	**0.0016**
**Blood culture positive**	5 (9.6)	0 (0.0)	0.5816
**Laboratory tests**			
White blood cells (*10^9/L)	11.6 (7.7, 15.8)	7.4 (5.3, 8.0)	**0.0044**
Neutrophils (*10^9/L)	10.2 (6.3, 14.1)	4.3 (3.2, 6.0)	**0.0004**
Lymphocytes (*10^9/L)	0.8 (0.5, 1.8)	1.3 (0.9, 1.6)	0.1604
Hemoglobin (g/L)	102 (84, 129)	134 (125, 143)	**0.0013**
Platelet (*10^9/L)	192 (128, 250)	176 (138, 296)	0.7380
K^+^ (mmol/L)	4.2 (3.8, 4.5)	4.1 (3.6, 4.5)	0.6197
Creatinine (mg/dL)	68.9 (47.9, 115.2)	66.8 (51.1, 80.2)	0.5254
BUN (mg/dL)	8.8 (6.1, 18.9)	5.8 (4.5, 8.7)	**0.0172**
CRP (mg/L)	85.9 (33.8, 168.5)	6.9 (2.8, 81.1)	**0.0101**
PCT (ng/ml)	0.4 (0.1, 1.9)	0.1 (0.1, 0.1)	**0.0004**

Abbreviation: asLRTI, acute severe lower respiratoty tract infection; BUN, Serum urea nitrogen; COPD, Chronic Obstructive Pulmonary Disease; CRP, C reactive protein; PSI, Pneumonia severity index; PCT, Procalcitonin.

* Continuous numerical data is shown as median (interquartile range) in this table, and categorical data are presented as n (%).The Mann Whitney test is used for comparison of continuous numerical data, while the Fisher's exact test is generally used for group comparison of categorical data. *p* <0.05 is defined as statistically significant.

**FIGURE 2:**
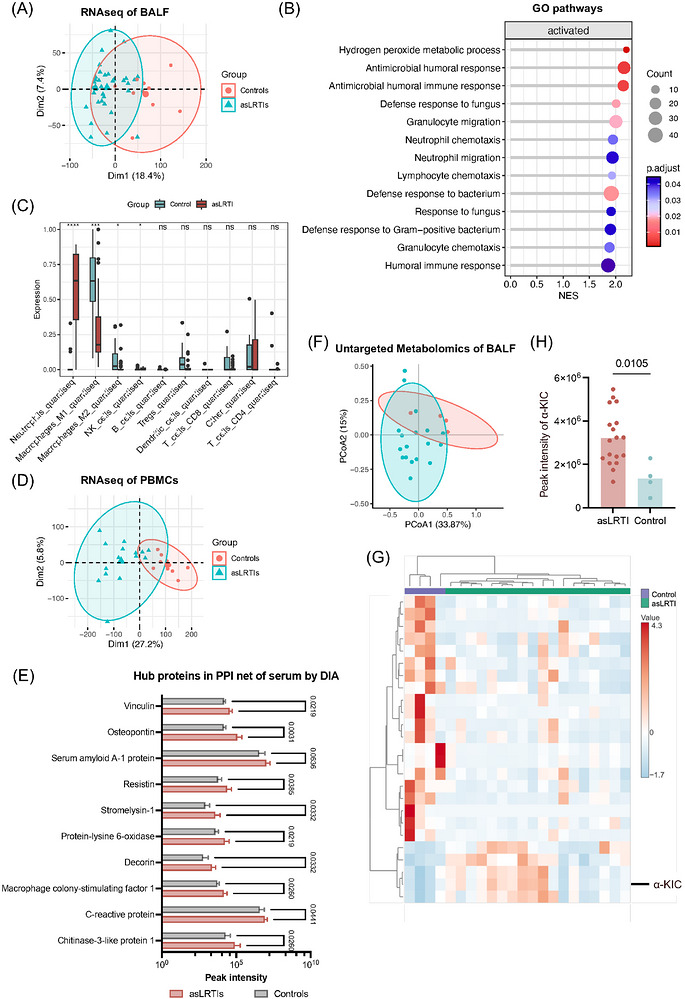
Landscape of lower respiratory tract in asLRTIs. (A) PCA plot on RNAseq of BALF sediment. (B) Enrichment of GO pathways–biological process by upregulated DEGs. (C) Immune infiltration by quantiseq algorithm on gene expression profiles. (D) PCA plot on RNAseq of PBMCs. (E) Expression of hub proteins screened by PPI on DIA of serum. (F) PCoA plot on untargeted metabolomics of BALF. (G) Heatmap of DAMs levels screened by VIP. (H) The levels of 𝛼‐KIC in asLRTIs and control. *p* value was calculated by Mann–Whitney test. *p* < 0.05 was defined as significant. Abbreviations: 𝛼‐KIC, α‐ketoisocaproic acid; DAMs, differentially abundant metabolites; DEGs, differentially expressed genes; GO, gene ontology; PCA, principal component analysis; PCoA, principal coordinate analysis; PPI, protein–protein interactive network analysis; VIP, variable importance for the projection.

Then we screened the gene expression profiles of BALF sediment from 41 asLRTIs and 16 controls (Figure 1B and Table S1). The principal component analysis (PCA) plot showed a trend toward separation between asLRTIs and controls (18.6% on Dimension 1) (Figure 2A), albeit with moderate overlap. Note that 1413 upregulated and 380 downregulated genes were screened in asLRTIs compared to controls (Figure S1B). Antimicrobial immune response and neutrophil‐related pathways were significantly enriched (Figure 2B). Consistently, neutrophils represented the predominant immune cell type with marked infiltration in asLRTIs (Figure 2C and Figure S1C). Importantly, the phenotype of neutrophil infiltration was validated by proteome of BALF, where we screened 494 upregulated proteins and enriched neutrophil‐related pathways in Gene On tology (GO) database significantly (Figure 1B and Figure S1D–F) (Table S1).

Although some overlap was observed, they were largely separable along Dim1 (27.2%) (Figure 2D) in PCA plot of gene expression data of peripheral blood mononuclear cells (PBMCs) from 15 asLRTIs and 10 controls (Figure 1B and Table S1). Following screening DEGs by adjusted *p* < 0.05 and |Log2FoldChange| > 3, “Neutrophil activation” was significantly enriched by upregulated DEGs (Figure S2A,B), which was consistent with the elevated count of neutrophils in peripheral blood (Table 1). Moreover, we also screened differentially expressed proteins (DEPs) of serum by DIA and observed distinct distributions through principal coordinate analysis (PCoA) (Figure 1B and Figure S2C) (Table S1). Acute‐phase response and acute inflammatory response were significantly enriched by upregulated DEPs (Figure S2D). Similarly, hub proteins, screened by protein–protein interaction (PPI) network, were notably elevated in asLRTI, including acute‐phase response protein like CRP, serum amyloid A‐1 protein (SAA1), chitinase‐3‐like protein 1 (CHI3L1), and resistin (RETN), which supported the pathways enriched (Figure 2E and Figure S2E,F).

In order to explore the overview of metabolite profile in asLRTI, we performed untargeted metabolomics on BALF from 18 asLRTI and 4 controls and targeted SCFAs on 14 asLRTI and 5 controls (Figure 1B and Table S1). A total of 434 metabolites were identified by untargeted metabolomics in this study. After normalization, we observed that the metabolite composition in BALF from asLRTIs was obviously different from controls (Figure 2F). We screened the top 25 metabolites that were differentially enriched in BALF based on the variable importance for the projection (VIP) value. Five components increased in the asLRTI group, while 20 components decreased compared to the controls (Figure 2G). We conducted significance analysis of metabolites (SAM) and identified α‐ketoisocaproic acid (α‐KIC) as a candidate biomarker, which was significantly elevated in asLRTI (Mann–Whitney *U* test, *p* = 0.0105, fold change [FC] = 2.7) (Figure 2H). Taken together, these findings supported that asLRTIs could be characterized as a hyperinflammatory state dominated by neutrophilic infiltration and systemic acute‐phase response, alongside profound dysbiosis of LRTM and related metabolites.

### 2.2 Characteristics Associated With Prognosis in asLRTIs

**FIGURE 3:**
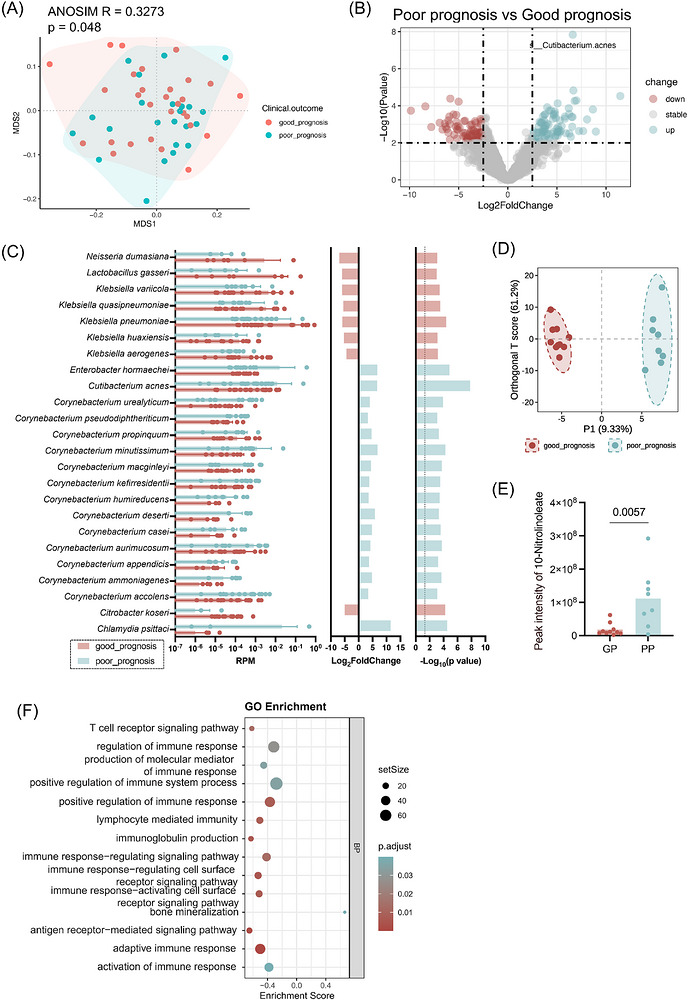
Characteristics associated with prognosis in asLRTIs. (A) asLRTI with different prognosis presented differentiated LRTM (*p* = 0.048). (B) Species with different abundance related to prognosis were identified by edgeR (log2FoldChange = 2.5 and *p* value < 0.01). (C) Top species related to prognosis in asLRTI were shown, including reads per million (RPM), log2FoldChange, and *p* value. (D) The distribution of metabolites in BALF identified by outcomes. (E) 10‐Nitrolinoleate was elevated in PP. (F) Enrichment of GO pathways–biological process by DEGs from RNAseq of BALF sediment. *p* value was calculated by Mann–Whitney test. *p* < 0.05 was defined as significant. GP, good prognosis; PP, poor prognosis.

Then we explored the features of LRTM in asLRTIs in relation to prognosis. Patients with asLRTI were divided into poor prognosis (PP) and good prognosis (GP) groups based on their prognosis within 100 days. Although no significant difference was observed in the alpha diversity indexes (Figure S3A), we discovered a significant difference in the distribution of LRTM between the PP and GP groups (ANOSIM *R* = 0.3273, *p* = 0.048) (Figure 3A). Using differental abundance analysis, 85 expanded species in the PP and 78 expanded species in the GP were identified (Figure 3B). We screened 24 species which were thought to locally infect or colonize the human respiratory tract, including species in the genera *Neisseria, Lactobacillus, Klebsiella, Enterobacter, Cutibacterium, Corynebacterium, Citrobacter*, and *Chlamydia* (Figure 3C). *Neisseria, Lactobacillus, Klebsiella*, and *Citrobacter* expanded in GP, whereas *Enterobacter, Cutibacterium, Corynebacterium*, and *Chlamydia* expanded in PP (Figure 3C). These results suggest an association between LRTM and the prognosis of patients with asLRTI. Interestingly, we also observed a complete separation of the distributions of metabolites in BALF identified by outcomes (Figure 3D). We screened top differentially abundant metabolites (DAMs) and identified that 10‐nitrolinoleate was noticeably elevated in PP (Figure 3E and Figure S3B).

Then we described the immune landscape of asLRTIs with different outcomes. The enrichment of adaptive immune response–related pathway by DEGs was notably decreased in gene expression profiles of both local and systemic conditions (Figure 3F and Figure S4A–C). Besides, we also described the clinical features of asLRTIs identified by outcomes. Only counts of lymphocytes was significantly decreased in PP (*p* = 0.0023), which is consistent with GO pathways enriched, whereas no significant difference was observed in PSI (Table S2). Unfortunately, too much overlap was found between the distributions of proteins in BALF identified by outcomes (Figure S4D). Due to the limited sample size, a subtle trend was observed in the distribution of proteins from serum, but might be masked by substantial variability (Figure S4E). Totally, an integrated framework combining LRTM, related metabolites, and the local immune landscape offers a potential approach to delineate the phenotype of asLRTIs.

### 2.3 Clustering of LRTM Characteristics in asLRTI

**FIGURE 4:**
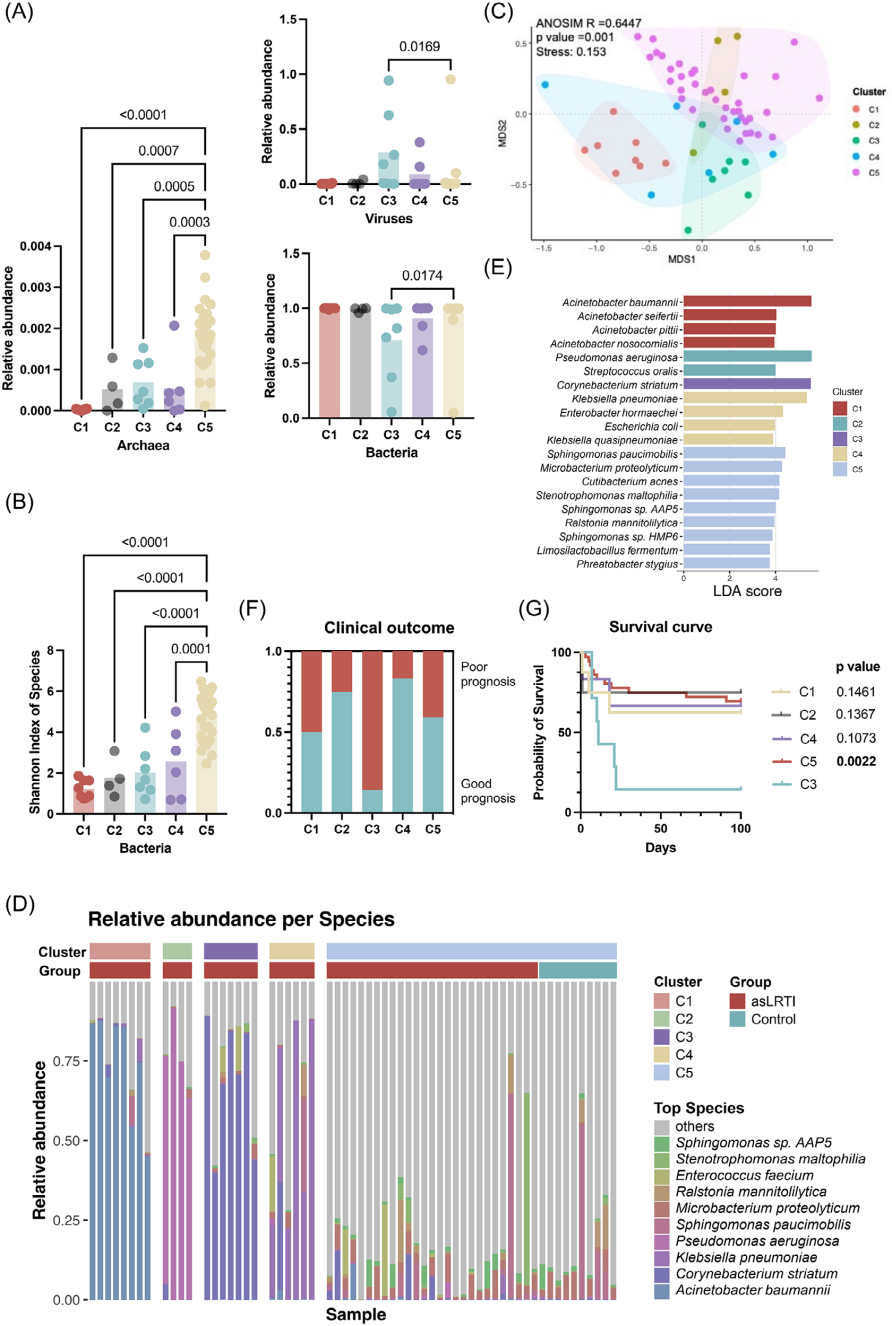
Clustering of LRTM characteristics in asLRTI. (A) Relative abundance of kingdoms in LRTM of clusters. (B) Shannon index of species in bacteria of each clusters. (C) Distribution of species in clusters. (D) Cumulative bar chart of relative abundance of species. (E) Different species in clusters identified by linear discriminant analysis effect size. (F) The percentage of poor prognosis in clusters. (G) Survival curves for C3 and other clusters. *p* value was calculated by two‐way ANOVA, and Holm–Sidák's multiple comparisons test was applied for post‐hoc test. **p < 0.01; ***p < 0.001; ****p < 0.0001.

To investigate the characteristics of LRTM in asLRTIs, we evaluated the number of clusters closest to the true cluster using contour coefficients (*n* = 5) (Figure S5A), and performed hierarchical clustering (C1–C5) based on the Bray–Curtis distance calculation (Figure S5B). It was observed that C3 was present with a notably elevated relative abundance of virus, while C5 was associated with a higher relative abundance of archaea (*p* < 0.05) (Figure 4A).

We then focused on the features of the bacteria because they were the major components of LRTM in nearly all patients. We calculated the Shannon index of species in bacteria and observed a significant decrease in clusters C1, C2, C3, and C4 (*p* < 0.05) (Figure 4B). There was a notable difference in the distribution of bacteria among the five clusters (ANOSIM *R* = 0.6447, *p* = 0.001) (Figure 4C and Figure S5C). The features of C5 seemed to be similar to those of the controls because nearly all memberships did not contain extremely expanded species (Figure 4D). We identified expanded species in other clusters such as *Acinetobacter baumannii* in C1, *Pseudomonas aeruginosa* in C2, *Corynebacterium striatum* in C3, and *Klebsiella pneumoniae* in C4 (Figure 4D). We could also observe that *Streptococcus oralis* was marked in C2, while *Enterobacter hormaechei* and *Escherichia coli* were identified in C4 (Figure 4E). Several species, which might be symbiotic microbiota in human, were presented in C5, such as *Sphingomonas paucimobilis*, *Microbacterium proteolyticum*, *Cutibacterium acnes*, *Stenotrophomonas maltophilia*, *Ralstonia mannitolilytica*, *Limosilactobacillus fermentum*, and *Phreatobacter stygius* (Figure 4E).

### 2.4 Clinical Phenotypes of asLRTIs Identified by Clustering

We describe basic demographic and clinical profiles of five clusters in Table S3. The rate of BALF culture positive was significantly decreased in C5 (40.7%), while the others exceeded 80% (Table S3), which might contribute to the characteristic expansion of pathogenic bacteria (Figure 4D). There seemed to be a higher count of lymphocytes in C2 and C4 compared to C1, C3, and C5, while a higher level of CRP was observed in C5, although it did not reach statistical significance (Table S3). Although the characteristics of the bacteria in LRTM were notably different, a relatively higher percentage of PP was observed in C1 and C5 than in C2 and C4 (Figure 4E,F). It is worth noting that patients in LRTM Cluster 3 exhibited the highest proportion of PP, with significantly reduced 100‐day survival probability (C3 vs. C5, *p* = 0.0022) (Figure 4F,G). Multivariate Cox regression, adjusted for key clinical confounders including age, PSI, and comorbidities, further confirmed that Cluster 3 remained an independent risk factor for adverse outcomes (adjusted HR = 4.182, 95% CI: 1.318–13.27, *p* = 0.0152) (Figure S5D). In line with the elevated level of virus abundance in C3 (Figure 4A), we found a positive relationship between *Corynebacterium striatum* and virus (Pearson *r* = 0.7867, *p* < 0.0001) (Figure S6A). We hypothesized that the expansion of virus might be accompanied by the proliferation of *Corynebacterium striatum*, leading to a high risk of PP. The alpha diversity of virus on phylum showed a noticeably decreased Shannon and Simpson index in C3 compared to C5 (*p* = 0.0320 and 0.0285) (Figure S6B). The expansion of *Peploviricota* was observed to be a feature for C3 (Figure S6C,D). Kaplan–Meier survival analysis showed a trend toward worse overall survival in patients with high *Peploviricota* abundance compared with those with low abundance (log‐rank test, *p* = 0.18) (Figure S6E), while no difference was observed in groups defined by virus relative abundance (*p* = 0.84) (Figure S6F). In multivariate Cox proportional hazards regression analysis, higher relative abundance of *Peploviricota* remained associated with an increased risk of death after adjustment for age, gender, PSI score, and comorbidity status (HR = 1.010, 95% CI: 0.998–1.022, *p* = 0.110) (Figure S6G). Although it did not reach statistically significant level, this consistent trend across all models suggests a potential prognostic role of *Peploviricota* in this cohort. These results reveal a trend toward poorer clinical outcomes in LRTM Cluster 3 relative to Cluster 5, independent of common clinical confounding factors.

### 2.5 Local Host Immune Response in Clusters of asLRTI

**FIGURE 5:**
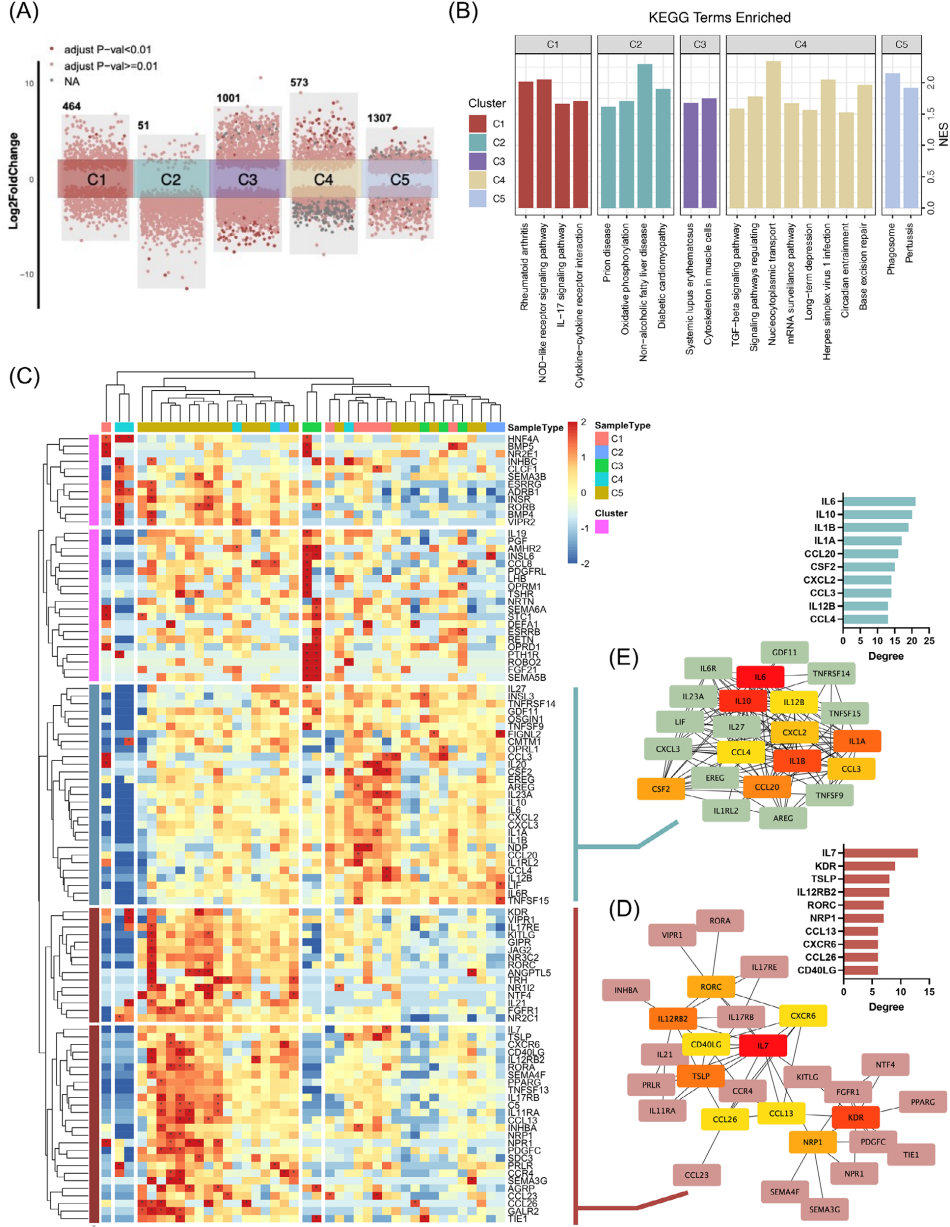
Local host immune response in clusters of asLRTI. (A) Multi‐volcano of each cluster for DEGs analysis in gene expression profiles. (B) KEGG pathway enriched by DEGs using method of GSEA. (C) Cytokines, chemokines, and their receptors in DEGs of clusters. (D) Protein–protein interaction (PPI) network and degrees in net of upregulated cytokines in C5. (E) PPI network and degrees in net of upregulated cytokines in C1.

To understand the interaction between LRTM and the host immune system, we studied the gene expression profiles of local infiltrating cells in BALF from asLRTIs. Compared to other clusters, 464 genes in C1, 51 genes in C2, 1001 genes in C3, 573 genes in C4, and 1307 genes in C5 were upregulated, while more regulated genes with adjusted *p* value < 0.01 were found in C4 (Figure 5A).

To explore the biological functions of the DEGs, we enriched pathways in the Kyoto Encyclopedia of Genes and Genomes (KEGG) and GO. “Systemic lupus erythematosus” was notably enriched in C3, indicating that C3 is characterized by interferon stimulation caused by viral infection (Figure 5B). KEGG pathways, including “Rheumatoid arthritis,” “NOD‐like receptor signaling pathway,” “*IL17* signaling pathway,” and “Cytokine‐cytokine receptor interaction,” and GO pathway “Cytokine activity” were significantly enriched in C1 (Figure 5B and Figure S7). Immune‐related GO pathways were enriched in C5 while only “Phagosome” and “Pertussis” in KEGG were enriched (Figure 5B and Figure S7). No direct immune‐related pathways were enriched in C2 or C4 (Figure 5B and Figure S7).

To present the immune response under local asLRTI conditions, we listed all cytokines, chemokines, and their receptors in the DEGs of each cluster. We observed a specific upregulated cluster of cytokines in the C5 group, including *IL7, KDR, TSLP, IL12RB2, RORC, NRP1, CCL13, CXCR6, CCL26*, and *CD40LG* (Figure 5C). *IL7* was revealed to be a top hub gene in the PPI network of the upregulated cytokine clusters in C5, relying on the highest degree (Figure 5D). Another specific cluster of upregulated cytokines was observed in C1, which included *IL6, IL10, IL1B, IL1A, CCL20, CSF2, CXCL2, CCL3, IL12B*, and *CCL4* (Figure 5C). *IL6* was identified as the top hub gene in the PPI network of the upregulated cytokine clusters in the C1 group (Figure 5E). This may indicate that different immune responses under local conditions are associated with different clusters of LRTM.

### 2.6 Proteomics of the Local Condition in Clusters of asLRTI

**FIGURE 6:**
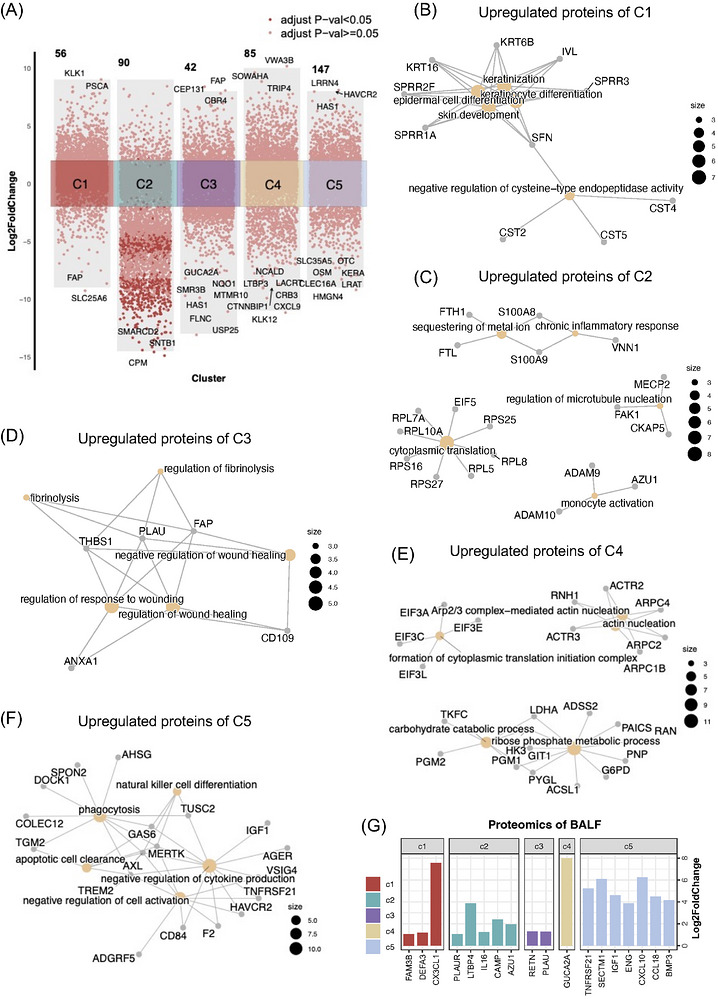
Proteomics of the local condition in clusters of asLRTI. (A) Different proteins expressed in each clusters. (B–F) GO pathways were enriched by upregulated proteins from each clusters. (G) Upregulated cytokines, chemokines, and their receptors in each clusters.

To describe the microenvironment of LRT, we analyzed protein expression profiles using DIA in BALF from asLRTIs. Compared to the other clusters, 56 proteins in C1, 90 proteins in C2, 42 proteins in C3, 85 proteins in C4, and 147 proteins in C5 were upregulated (Figure 6A). To study the function of the upregulated proteins, we enriched the pathways in the GO database. “Keratinization,” “Keratinocyte differentiation,” and “Epidermal cell differentiation” were enriched in C1, indicating the self‐protection of local epithelial cells in the LRT (Figure 6B). Innate immune system was considered as active in C2 in which “Monocyte activation” and “Chronic inflammatory response” were enriched, and S100A8, S100A9, ADAM10, ADAM9, and AZU1 were upregulated (Figure 6C). “Fibrinolysis” and “Regulation of wound healing” revealed that cell‐to‐cell and cell‐to‐matrix interactions might play a role in C3 (Figure 6D). Only metabolic process‐related pathways were enriched in the C4 group (Figure 6E). Positive immune response was shown in C5 and “Phagocytosis,” “Negative regulation of cytokine production,” “Negative regulation of cell activation,” and “Natural killer cell differentiation” were enriched (Figure 6F). Next, we screened the cytokines, chemokines, and their receptors that were upregulated in each cluster. CX3CL1, which is chemotactic for T cells and monocytes, was notably upregulated in C1, whereas CXCL10 and CCL18 were significantly elevated in C5 (Figure 6G). In addition, the expression of apoptotic pathway‐related proteins, including TNFRSF21, SECTM1, IGF1, and BMP3, was elevated in the C5 group (Figure 6G).

### 2.7 *Acinetobacter baumannii*–asLRTI Was Characterized by IL17 Signaling Pathway

**FIGURE 7:**
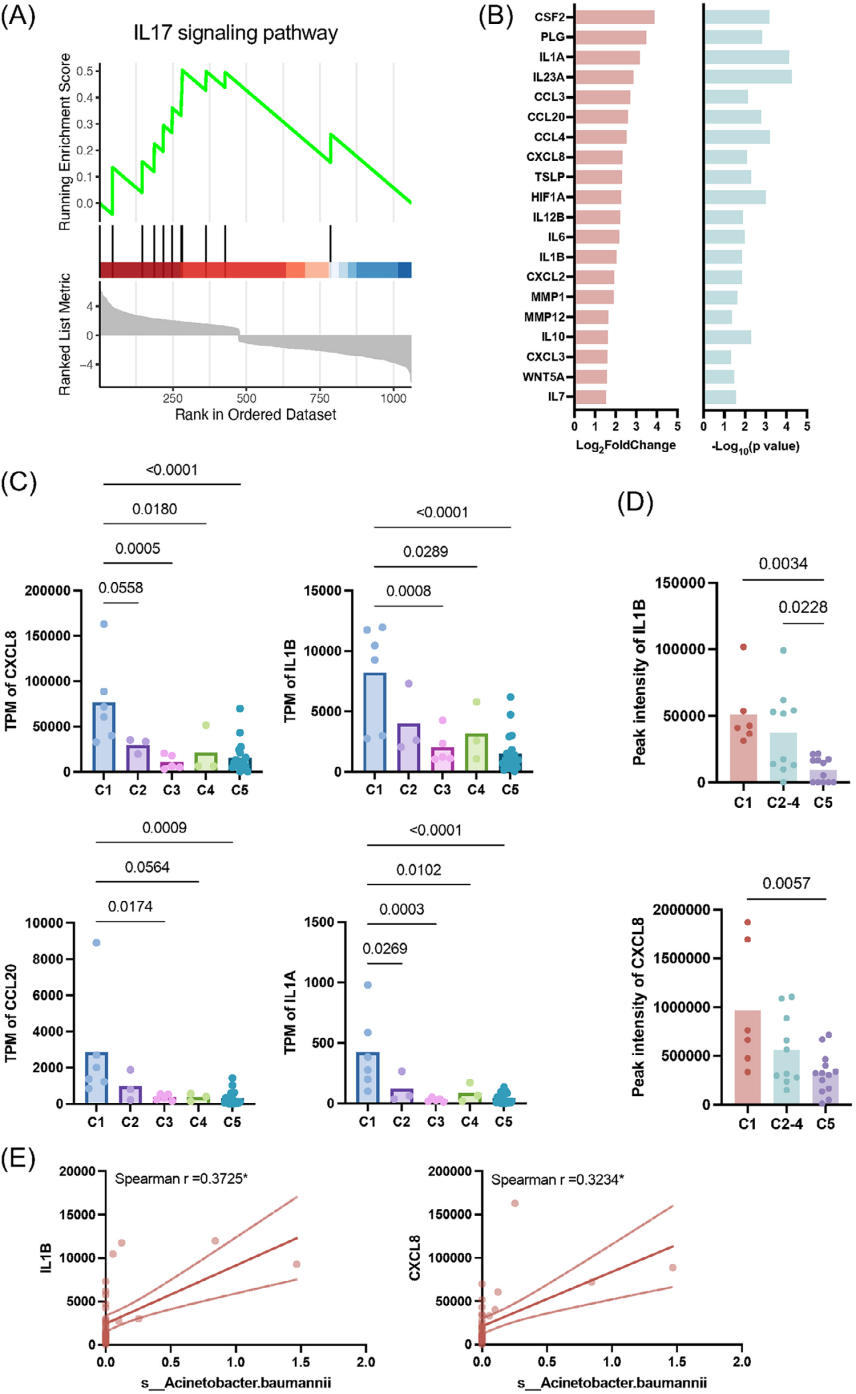
*Acinetobacter baumannii*–asLRTI was characterized by IL17 signaling pathway. (A) IL17 signaling pathway was significantly enriched in C1. (B) Relative expression of top hub genes in C1. (C) *CXCL8*, *IL1B*, *CCL20*, and *IL1A* were elevated in C1. (D) IL1b and CXCL8 were elevated in protein level. (E) Gene expression of *IL1B* and *CXCL8* were associated with RPM of *Acinetobacter baumannii*. *p* value was calculated by two‐way ANOVA, and Tukey's multiple comparisons test was applied for post‐hoc test. *p < 0.05; **p < 0.01; ***p < 0.001; ****p < 0.0001.

C1 was characterized as *Acinetobacter baumannii* that expanded in the LRTM. To explore the host immune response to *Acinetobacter baumannii*, we performed PPI analysis on the upregulated genes in C1 and identified hub genes through the degree in net (Figure S8). We observed that *IL17* signaling pathway was notably enriched in hub genes, such as *IL6, IL1B, CXCL8, IL1A, CCL20, CSF2, CXCL2*, and *MMP1* (Figure 7A and Figure S8). Hub gene expression was significantly elevated in the C1 group (Figure 7B). *CXCL8, IL1B, IL1A*, and *CCL20* were notably elevated in the C1 group, whereas the protein levels of IL1B and CXCL8 were increased (Figure 7C,D). In addition, there was a notable positive association between *Acinetobacter baumannii* and the expression of *IL1B* or *CXCL8* (Figure 7E). It suggested that *Acinetobacter baumannii–*asLRTI might be characterized by the activation of *IL17* signaling pathway in host immune response, compared to the other clusters.

## 3 Discussion

Our study described the features of LRTM in patients with asLRTIs and identified five clusters based on the bacterial contribution of LRTM. Local conditions, including LRTM and the host immune response, have a significant impact on the prognosis of asLRTIs. A subclass dominated by the expansion of *Acinetobacter baumannii* was also identified, characterized by the activation of the host *IL‐17* signaling pathway. Our results demonstrated a correlation between LRTM and asLRTI prognosis. These data suggest that LRTM may be a notable feature of asLRTIs.

mNGS has gradually been applied to the diagnosis of infectious diseases because of its unbiased detection of pathogens [12]. Several reports have described the diagnostic value of mNGS for LRTI [13, 14, 15, 16, 17, 18, 19, 20, 21, 22, 23, 24, 25]. Fourgeaud et al. systematically evaluated the clinical diagnostic performance of metagenomics and demonstrated the potential benefits of adding mNGS to routine diagnostic workflows to detect and discover rare or novel pathogens [26]. LRTI is not a disease caused by a single microorganism but is a result of the interaction between LRTM and the host immune response [5]. In our study, we made mNGS a powerful and multifunctional tool for studying LRTM and supplemented the clinical application of mNGS in asLRTIs.

Five clusters that depend on the LRTM were identified in our study. Cluster C3, with virus expanded was significantly related to the PP of asLRTIs. Interestingly, *Corynebacterium striatum* was found to expand in C3, with a positive correlation with the relative abundance of HSV‐1. *Corynebacterium striatum* is a normal parasitic bacterium in the skin and nasopharyngeal mucosa and an opportunistic pathogen in hospitals [27]. It is the main cause of HAP in severe cases, with a mortality rate similar to that of methicillin‐resistant *Staphylococcus aureus* infections [28]. Our observations mirror those of other studies that clustered with *Corynebacterium striatum* expanded, related to a notably high mortality in 100 days. In addition, the association between *Corynebacterium striatum* and HSV‐1 might suggest cooperation between virus and bacteria that promotes the mortality of asLRTIs. More consideration should be given to the LRTMs of asLRTIs and their cooperation with virus, archaea, and bacteria.

Although LRTM and the host immune response in local and systemic conditions of asLRTI were significantly different from controls, we considered local conditions to be crucial in the prognosis. Previous studies have revealed that specific species in LRTM can lead to the release of pro‐inflammatory cytokines such as IL‐8 and TNF‐α  [27, 28, 29, 30]. Chen et al. identified an interaction between LRTM and inflammatory pathways in the host that potentially influenced the onset and progression of LRTIs [5]. In this study, we focused on LRTM clusters and their related local conditions. For the expansion of the virus, C3 was presented as a characteristic of IFN stimulation, which is consistent with previous studies [31]. Although no clear pathogen was characterized for C5, we observed an upregulated cytokine net with hub genes, such as *IL7*, *KDR*, *TSLP*, *IL12RB2*, *RORC*, and *NRP1*.

In our study, C1 clustered with the significantly expanded features of *Acinetobacter baumannii*. We observed upregulation of the IL17 signaling pathway in C1, which is consistent with previous studies where *Acinetobacter baumannii* adheres to and activates neutrophils, induces an increase in IL‐8 production, and promotes the migration and transport of *Acinetobacter baumannii* with neutrophils [32, 33, 34]. *CXCL8* and *IL1B* were notably elevated in C1 and were associated with the RPM of *Acinetobacter baumannii*. Unexpectedly, proteins upregulated in C1 were enriched in self‐protection pathways of local epithelial cells, such as “Keratinization” and “Epidermal cell differentiation.” This might explain the relationship between a clear pathogen and PP of asLRTIs. Further investigations should be planned in a larger cohort to investigate the interaction between LRTM and local conditions and their roles in the progression of asLRTIs.

However, this study had certain limitations. First, the small number of samples enrolled in the study resulted in too few samples in the subcategories after LRTM clustering, which could have easily led to bias in the analysis. A primary limitation of this study lies in the scale of the metabolomic analysis. The profiling was performed on a relatively small subset of the cohort (*n* = 18 for asLRTIs and *n* = 4 for controls). The same applies to DIA on serum and RNAseq on PBMCs. This sample size critically limits the statistical power to detect subtle differences and affects the stability and generalizability of the results. Consequently, the metabolomic findings, including the identification of candidate biomarkers such as α‐KIC, should be interpreted as preliminary and hypothesis‐generating. They underscore biologically plausible pathways but require independent validation in a larger, well‐powered cohort. Future studies designed with a prior sample size calculation are essential to definitively characterize the metabolic phenotype of asLRTIs and its integration with the microbiome data. Besides, our analysis was limited by cohort size in its ability to perform adequately powered multivariable modeling. While intriguing associations were observed, confirming LRTM as an independent predictor of prognosis will require validation in larger, dedicated studies. Such efforts are strongly justified to translate these microbial insights into clinically actionable knowledge. An inherent challenge in this study is the imbalance in sample size between the asLRTI patient group (*n* = 52) and the control group (*n* = 10). This disparity arises from the practical and ethical constraints of obtaining BALF samples. Bronchoscopy is an invasive procedure performed only with a strict clinical indication. Our control samples were derived from patients with mild interstitial pneumonia who underwent the procedure for diagnostic purposes. Recruiting a large cohort of such control subjects without active infection is fundamentally difficult, reflecting a common limitation in intensive phenotyping studies of severe infectious diseases. This imbalance can reduce the statistical power for between‐group comparisons. To address this, we employed statistical methods robust to unequal group sizes, including nonparametric tests and permutation‐based approaches for multivariate analyses. Notably, our metagenomic approach was limited to DNA sequencing, which precludes the detection of RNA viruses and non‐DNA viral taxa. Further dedicated virome analysis in prospective cohorts will help better clarify the interplay between viral signatures and patient prognosis. Next, there was no restriction on the causes of asLRTIs in the enrollment criteria, and various types of pneumonia, such as community‐acquired pneumonia, aspiration pneumonia, and ventilator‐associated pneumonia, were included, which increased heterogeneity and difficulty in the analysis and weakened the analysis of specific causes of LRTM dysregulation. In addition, because of the uniqueness of LRTM in humans, this study only analyzed LRTM in asLRTIs at the population cohort level and did not reproduce it at the animal model level. In the future, it will be necessary to validate this hypothesis in larger populations from different sources, and to verify the interaction between LRTM and host conditions at the molecular, cellular, and animal levels.

In conclusion, our study identified five clusters of asLRTIs based on the features of LRTM and described their microbial content, local conditions, and related clinical prognosis. Local conditions are considered crucial and are influenced by LRTM in the progression of asLRTI. The prognosis of *Acinetobacter baumannii*–asLRTI was affected by both IL17 signaling pathway and self‐protection of local epithelial cells, indicating complexity of interaction between LRTM and host local conditions. Overall, LRTM analysis should be considered in the diagnostic process of asLRTIs to supplement the clinical utility of mNGS.

## 4 Materials and Methods

### 4.1 Study Design

In this study, patients with asLRTI admitted to the China–Japan Friendship Hospital and Beijing Chaoyang Hospital from January 2022 to March 2023, were recruited as research subjects. The inclusion criteria [35]: (a) age > 18 years, (b) acute disease course with a duration not exceeding 1 month, and (c) meeting the diagnostic criteria for asLRTIs. As a previous article described [36], meeting one primary criterion or no less than three secondary criteria was considered as asLRTI—(a) primary criterion: invasive mechanical ventilation, infectious shock requiring vasopressors; (b) secondary criteria: respiratory rate > 30 beats per minute, oxygenation index ≤ 250 mmHg, multiple lung lobe infiltration, consciousness disorders and/or orientation disorders, uremia (serum urea nitrogen ≥ 20 mg/dL), decreased white blood cells (white blood cell count < 4 × 10^9^/L), thrombocytopenia (platelet count < 100 × 10^9^/L), low body temperature (below 36°C), low blood pressure (requires active fluid resuscitation). Exclusion criteria: HIV patients; malignant hematological diseases and other immunodeficient patients (hematological or solid tumor patients receiving chemotherapy within 3 months; solid organ or bone marrow transplantation; graft‐versus‐host disease; use of immunosuppressive drugs or prednisone ≥ 20 mg/day (or equivalent dose of other hormones) during onset for more than 30 days; simultaneously infected with HIV, peripheral blood T cell subsets CD4 cell count < 200/mm^3^; primary/hereditary immunodeficiency disease; diagnosed as COVID‐19 positive pneumonia patients. Patients with mild or asymptomatic interstitial lung disease admitted during the same period were selected as controls. Clinical samples, including serum and EDTA anticoagulant for peripheral blood, were collected within 24 h after admission. Clinical baseline data were collected on the day of admission for BALF collection, including vital signs, medical history, laboratory tests, and prognostic information. The severity of the pneumonia was evaluated and recorded by a respiratory specialist upon admission [37]. Patients were followed up for 100 days after admission. GP is defined as significant relief of respiratory symptoms in patients upon completion of follow‐up, transfer from ICU to general ward, or discharge after recovery. PP is defined as all‐cause death during the follow‐up period, or the situation where symptoms do not improve and cannot be transferred out of the ICU by the end of the follow‐up period.

### 4.2 Sample Collection

As previously described [38], BALF was collected within a week of hospitalization during a nasal bronchoscopy examination. Following centrifugation at 600 *g* for 5 min, supernatant was isolated and stored at −80°C for mNGS, DIA, and nontargeted metabolomics detection. Cell sediment was fully lysed in TRIzol (Invitrogen), and stored at −80°C for RNA extraction. Collected fresh EDTA anticoagulated peripheral blood on the same day, and isolated PBMCs in 4 h using Ficoll‐Paque PLUS (GE Healthcare) according to the instruction of manufacturer. After washing once with PBS, PBMCs were lysed in TRIzol (Invitrogen) for RNAseq.

### 4.3 mNGS Library Preparation, Sequencing, and Bioinformatics Analysis

QIAamp DNA Microbiome Kit (QIAGEN) was used to purify and enrich bacterial microbiome DNA from BALF, effectively removing host DNA. After confirming the purity and integrity of DNA, sequencing libraries were generated on a total amount of 1 µg DNA per sample using a NEBNext Ultra DNA Library Prep Kit for Illumina (NEB). Following assessment of the insert size of library quality on an Agilent 2100 Bioanalyzer, library pools were loaded onto an Illumina NovaSeq 6000 sequencing system for paired‐end 150 bp sequencing to generate approximately 120 million reads for each library. Four reagent controls (DNA extraction blanks) were included for sequencing.

Readfq (https://github.com/cjfields/readfq) was performed to obtain clean data for subsequent analysis through (a) removing reads containing over 40 bp of low‐quality bases (Phred score ≤ 38), (b) discarding reads with N bases exceeding 10 bp, and (c) eliminating reads overlapping with adapters by more than 15 bp. To address potential host contamination, the clean data are aligned against a host reference database using Bowtie2 [39] (parameters: –end‐to‐end –sensitive ‐I 200 ‐X 400) to filter out host‐derived reads. A kraken2‐bracken analysis [40, 41] process was used for alignment and annotation of unigenes sequences with those of bacteria, fungi, archaea, and viruses extracted from NCBI's NR database (https://www.ncbi.nlm.nih.gov/), including one human (hg38), 185,630 bacterial, 308 archaeal, 54 fungal, 9021 viral, 178 invertebrate, and 39 protozoan reference genomes. LCA algorithm (https://en.wikipedia.org/wiki/Lowest_common_ancestor) was adopted to determine the species annotation information of the sequence [42]. Out of the results of LCA annotation and gene abundance table, the abundance of each sample at each taxonomy (kingdom, phylum, class, order, family, genus, or species) was acquired. The abundance of a species in a sample is equal to the sum of the abundance of those genes annotated as that species [21, 43, 44]. The number of genes of a species in a sample is equal to the number of genes whose abundance is nonzero among the genes annotated as that species. Relative abundance was calculated and visualized by ggpubr package. Shannon index, Simpson index, Richness index, and Pielou index were used to present the alpha diversity of species by vegan package (https://github.com/vegandevs/vegan). Beta diversity on species between groups was shown by PCoA based on Bray–Curtis's distance. Clusters were identified using *K*‐means clustering method and silhouette coefficient was calculated to evaluate clustering effectiveness. Nonmetric multidimensional scaling (NMDS) and ANOSIM were performed by vegan package to assess the differences between groups. ANOSIM *R* > 0 means the distance within the group is less than the distance between groups, indicating that the grouping is valid. Differentially abundant species between groups were identified using the linear discriminant analysis effect size (LEfSe) analysis (LDA score > = 4) [45]. EdgeR was performed to identify differentially abundant species between GP and PP groups with a cut‐off value of |log2FC| > 2.5 and *p* value < 0.01.

### 4.4 Bulk RNAseq Library Preparation, Sequencing, and Bioinformatics Analysis

The procedure of RNAseq was performed as previously described [38]. In brief, following total RNA extracted, the integrity was assessed using the RNA Nano 6000 Assay Kit of the Bioanalyzer 2100 system (Agilent Technologies). Following RNA sample preparations, library preparations were sequenced on an Illumina NovaSeq 6000 sequencing system and 150 bp paired‐end reads were generated. Clean reads were obtained by removing reads containing adapter, reads containing poly‐*N*, and low‐quality reads from raw data. Hisat2 (v2.2.1) [46] was used to align the clean reads to Genome Reference Consortium Human Build 38 (GRCh38). Following sorting by samtools (v1.5) [47, 48], gene counts were calculated by featureCounts (v2.0.1) [49]. DEGs were identified by DESeq2 (v1.44.0) [50], according to adjusted *p* value < 0.05 and |Log2FoldChange|> 1. FactoMineR and factoextra packages were performed for PCA. Gene set enrichment analysis (GSEA) was conducted to enrich functional pathways on KEGG database and GO database. Immune infiltration was conducted by IOBR package, which integrated several methods including MCPcounter and quantiseq [51]. Gene clusters were generated by hierarchical clustering based on the normalized expression levels of DEGs, as visualized by the dendrogram on the left side of the heatmap. Each cluster represented a distinct gene set with consistent upregulated or downregulated expression patterns across sample groups. Upregulated DEGs were loaded on the STRING tools (http://www.string‐db.org/) to conduct PPI analysis with a confidence of 0.400. Cytoscape (https://cytoscape.org) was used to calculate the degree, closeness, and betweenness of the net.

### 4.5 DIA Proteomics and Bioinformatics Analysis

Samples were solubilized in protein lysis buffer (8 M urea, 100 mM triethylammonium bicarbonate, pH = 8.5), clarified by centrifugation, and then reduced with dithiothreitol. The free cysteines were then alkylated with iodoacetamide in a dark incubation. Protein concentration was determined using a Bradford assay with a BSA standard curve (0–0.5 µg/µL), and 20 µg of each sample was verified by 12% SDS‐PAGE. Following tryptic digestion (4 h at 37°C, then overnight), peptides were acidified, desalted using C18 columns, and eluted with 70% acetonitrile/0.1% formic acid prior to lyophilization. Lyophilized peptides were reconstituted and separated on a Vanquish Neo UHPLC system using a C18 column with a programmed acetonitrile gradient. MS analysis was performed on an Orbitrap Astral mass spectrometer in DIA mode, with full MS scans at 240,000 resolutions and DIA scans at 80,000 resolutions. Raw data were collected and analyzed on DIA‐NN software (direct DIA mode), including spectral library searching and precursor quantification with iRT‐based retention time (RT) correction, applying a *Q*‐value cutoff of 0.01 and filtering to retain only peptide‐spectrum matches and proteins at a 1% false discovery rate (FDR). DEPs were defined as proteins with an adjusted *p* value < 0.05 and |log2Foldchange| > 1. Bioinformatic analyses and visualization, including volcano plot, GO/KEGG enrichment, and PPI network analysis, were performed by R.

### 4.6 Nontargeted Metabolomics and Bioinformatics Analysis

Nontargeted metabolic profiling was performed by using an ultra‐high performance liquid chromatography coupled to tandem mass spectrometry (UHPLC‐MS/MS) system (Vanquish Flex UHPLC‐TSQ Altis, Thermo Scientific), as previously described [38, 52]. Briefly, 100 µL BALF sample was prepared and finally loaded on UHPLC‐MS/MS for metabolomics analysis. The parameters of the ESI ion source were as follows: spray voltage 3500 V, sheath gas flow rate 35 psi, aux gas flow rate 10L/min, capillary temp 320°C, S‐lens RF level 60, aux gas heater temp 350°C, polarity positive and negative, and mass nucleus ratio (m/z) collection range 100–1500. Raw data were screened and filtered by Compound Discoverer 3.3 (Thermo Scientific) according to RT and m/z, followed by alignment to mzCloud (http://www.mzcloud.org/), mzVault, and Masslist database. Metabolites were identified and relatively quantified by standardized original quantitative result. Metabolomics were annotated by searching KEGG database (https://www.genome.jp/kegg/pathway.html), Human Metabolome Database (HMDB, https://hmdb.ca/metabolites), and lipid metabolites and pathways strategy (LIPIDMaps, http://www.lipidmaps.org/), and visualized on R. Multivariate statistical analysis section was conducted by metaX [53] to obtain the VIP values of each metabolite. SAM and orthogonal partial least squares discriminant analysis (OPLS‐DA) were conducted on MetaboAnalyst (https://www.metaboanalyst.ca) to show the differences in groups. The criteria for screening differential metabolites were VIP > 1, *p* value < 0.05, and FC ≥ 2 or FC ≤ 0.5.

### 4.7 Statistical Analysis

Statistical analysis and visualization were performed using R software or GraphPad Prism 9.0. The Mann–Whitney *U* test was used to compare continuous variables, and the Kruskal–Wallis (KW) test was used to evaluate the differences between groups. The *χ* ^2^ test or Fisher exact test was utilized for comparison of categorical variables. All statistical analyses were performed by SPSS 26.0 Statistical Software Package for the Social Sciences (SPSS Inc., Chicago, Illinois, USA).

## Author Contributions

**Minghua Zhan**: conceptualization, methodology, writing – original draft. **Hongbin Chen**: methodology, funding acquisition, writing – review and editing. **Ziyao Li**: investigation, validation, writing – review and editing. **Si Liu**: software, data curation, visualization. **Binghuai Lu**: formal analysis, resources, writing – review and editing. **Zhang Wang**: methodology, writing – review and editing. **Hui Wang**: data curation, writing – review and editing. All authors have read and approved the final manuscript.

## Funding

This study was funded by the National Key Research and Development Program of China (2022YFA1304300) and the Beijing Municipal Science and Technology Commission Program (Z191100006619100).

## Ethics Statement

This study was approved by the Institutional Ethics Review Committee of the Peking University People's Hospital (2019PHB134). Sample collection and medical record queries were approved by the Ethics Review Committees of the China–Japan Friendship Hospital (2021‐100‐K61) and Beijing Chaoyang Hospital (2023‐3‐10‐2). Informed consent was obtained from all patients prior to sample collection.

## Conflicts of Interest

The authors declare no conflicts of interest.

## Supporting information



mco270907‐supp‐0001‐SuppMat.pdf

## Data Availability

All data are available in the main text or in the Supporting Information. The data set supporting the results of this article has been deposited under the National Genomics Data Center with the BioProject identifier CRA026226.
