# Supplementary material for: Lower Respiratory Microbiome Dysbiosis Is Associated With Poor Prognosis in Acute Severe Lower Respiratory Tract Infection
Source: MedComm (2020). 2026 Aug 9;7(8):e70907. doi: 10.1002/mco2.70907 (PMC13454543; doi:10.1002/mco2.70907)
Supplement: Supplementary file 1 [file MCO2-7-e70907-s001.pdf]

Supplemental materials

Lower respiratory microbiome dysbiosis is associated with poor prognosis in acute severe lower respiratory tract infection

Authors: Zhan Minghua[1, 2, #], Chen Hongbin[1, #, *], Li Ziyao[3, #], Liu Si[1], Lu Binghuai[3], Wang Zhang[4, *], Wang Hui[1, *]

Affiliations:

[1] Department of Clinical Laboratory, Peking University People's Hospital, Beijing, China;

[2] Hebei Key Laboratory of Pathogenic Mechanisms and Diagnosis & Treatment Technologies for Lung Microbiome, Department of Clinical Laboratory, First Affiliated Hospital of Hebei North University, Zhangjiakou, Hebei, China;

[3] Laboratory of Clinical Microbiology and Infectious Diseases, Department of Pulmonary and Critical Care Medicine, Center of Respiratory Medicine, National Clinical Research Center for Respiratory Diseases, National Center for Respiratory Medicine, China-Japan Friendship Hospital, Beijing, China; Institute of Respiratory Medicine, Chinese Academy of Medical Sciences, Beijing, China;

[4] Institute of Ecological Sciences, School of Life Sciences, South China Normal University, Guangzhou, Guangdong Province, China

# Zhan Minghua, Chen Hongbin and Li Ziyao contributed equally to this article.

*** Corresponding author**: Wang Hui, Department of Clinical Laboratory, Peking University People's Hospital, Beijing 100044, China, whuibj@163.com; Chen Hongbin, Department of Clinical Laboratory, Peking University People's Hospital, Beijing 100044, China, chenhongbin_pkuph@163.com; Wang Zhang, Institute of Ecological Sciences, School of Life Sciences, South China Normal University, Guangzhou, Guangdong Province, 510631, China, wangz@m.scnu.edu.cn.

Figure S1

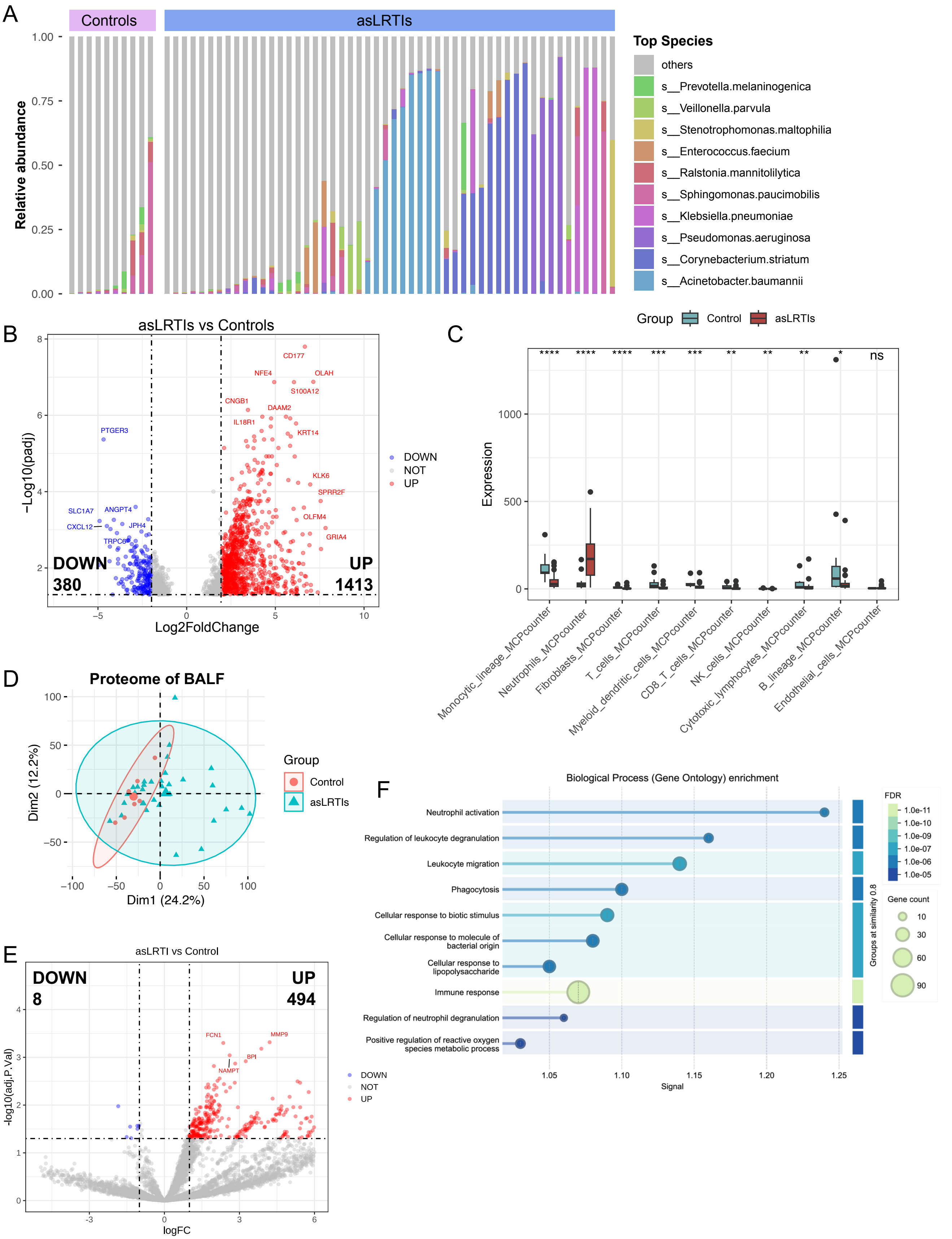

Figure S1 Local landscape in lower respiratory tract of asLRTIs in this study.
(A) The cumulative bar chart of relative abundance of LRTM; (B) Volcano plot of different expression genes by RNAseq in asLRTIs compared to control; (C) Immune cells infiltration analyzed by MCPcounter; (D) PCA plot of Proteome of BALF; (E) Volcano plot of different expression proteins by DIA in asLRTIs compared to control; (F) Enriched pathways in GO database were conducted on upregulated proteins.
Abbreviations: asLRTIs, acute severe lower respiratory tract infections; LRTM, lower respiratory tract microbiome; PCA, principal component analysis; DIA, Data-Independent Acquisition Proteomics; GO, gene ontology.

Figure S2

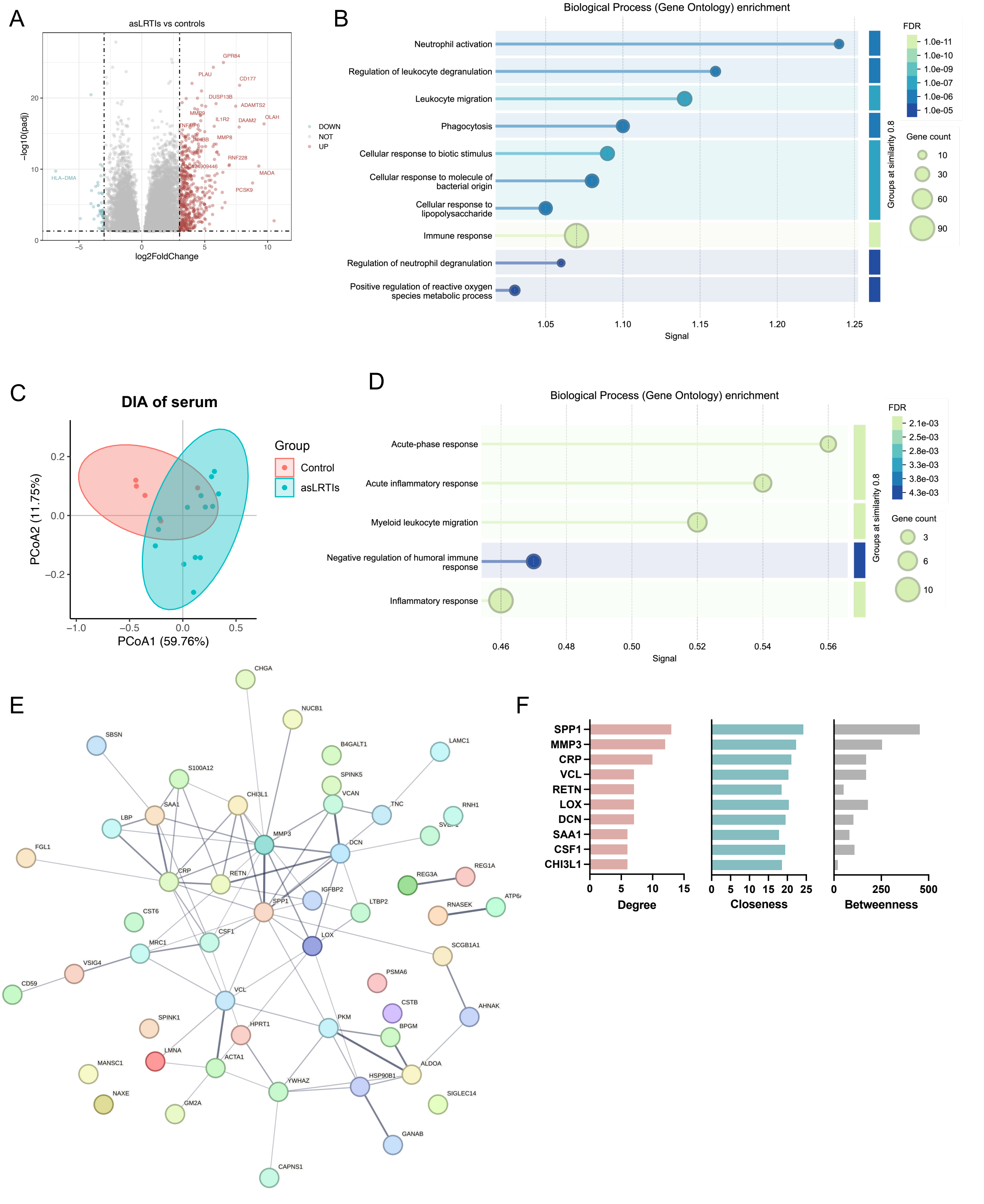

Figure S2 Systemic landscape in lower respiratory tract of asLRTIs in this study.
(A) Volcano plot of DEGs by RNAseq on PBMCs in asLRTIs compared to control; (B) Enriched pathways in GO database were conducted on upregulated DEGs; (C) PCoA plot of Proteome of serum; (D) Enriched pathways in GO database were conducted on upregulated DEPs; (E) PPI network by upregulated DEPs; (F) Parameters of hub proteins in PPI network.
Abbreviations: DEGs, different expression genes; PBMCs, Peripheral blood mononuclear cells; PCoA, Principal Coordinate Analysis; DEPs, different expression proteins; PPI, protein-protein interactive.

Figure S3

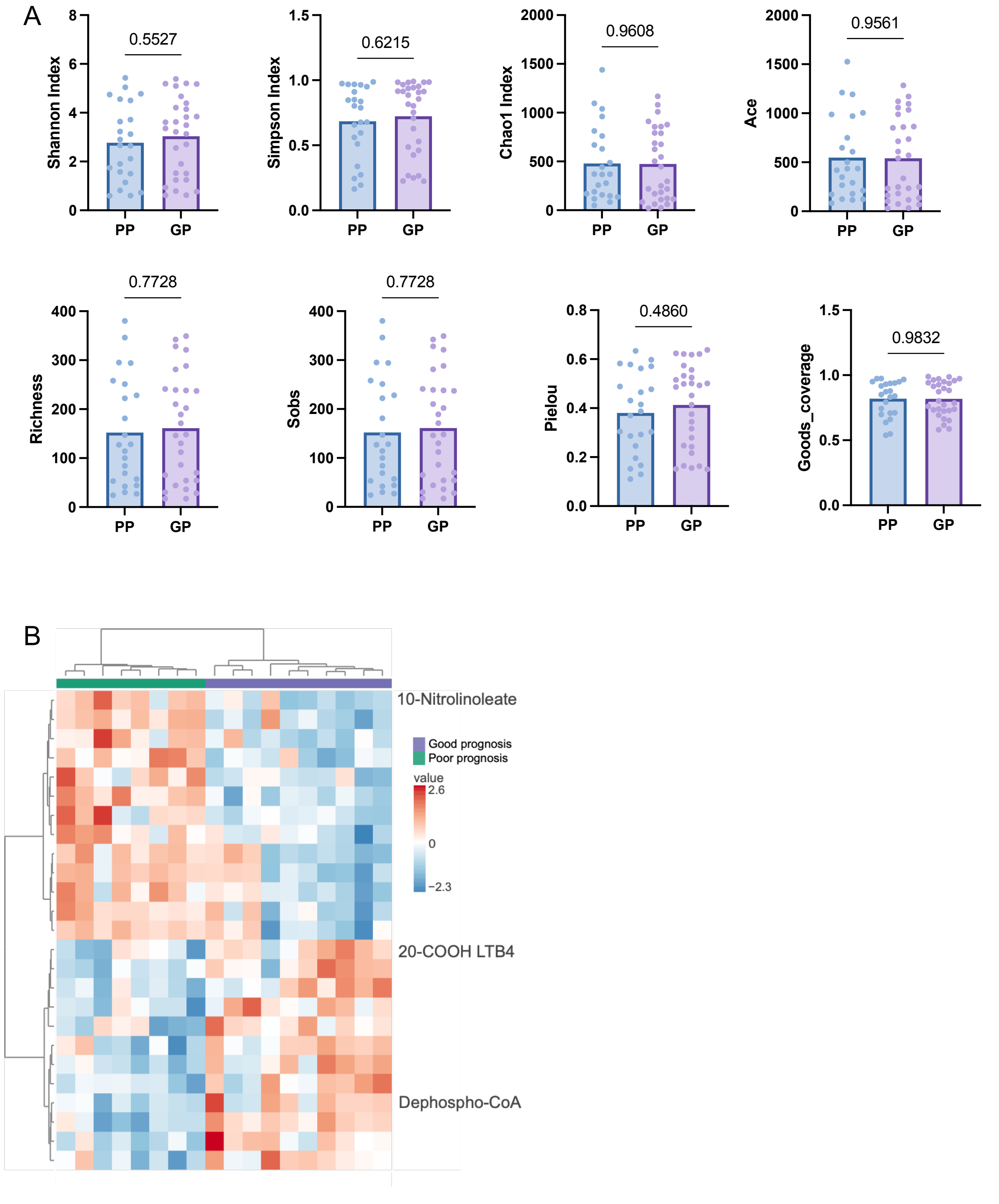

Figure S3 The features asLRTI with different outcomes.
(A) Alpha diversity index of LRTM in asLRTI with different prognosis; (B) Top 25 metabolites screened by VIP value in asLRTI vs control.
p value was calculated by T-test.
$p < 0.05$ was defined as significant.
Abbreviations: PP, poor prognosis; GP, good prognosis.

Figure S4

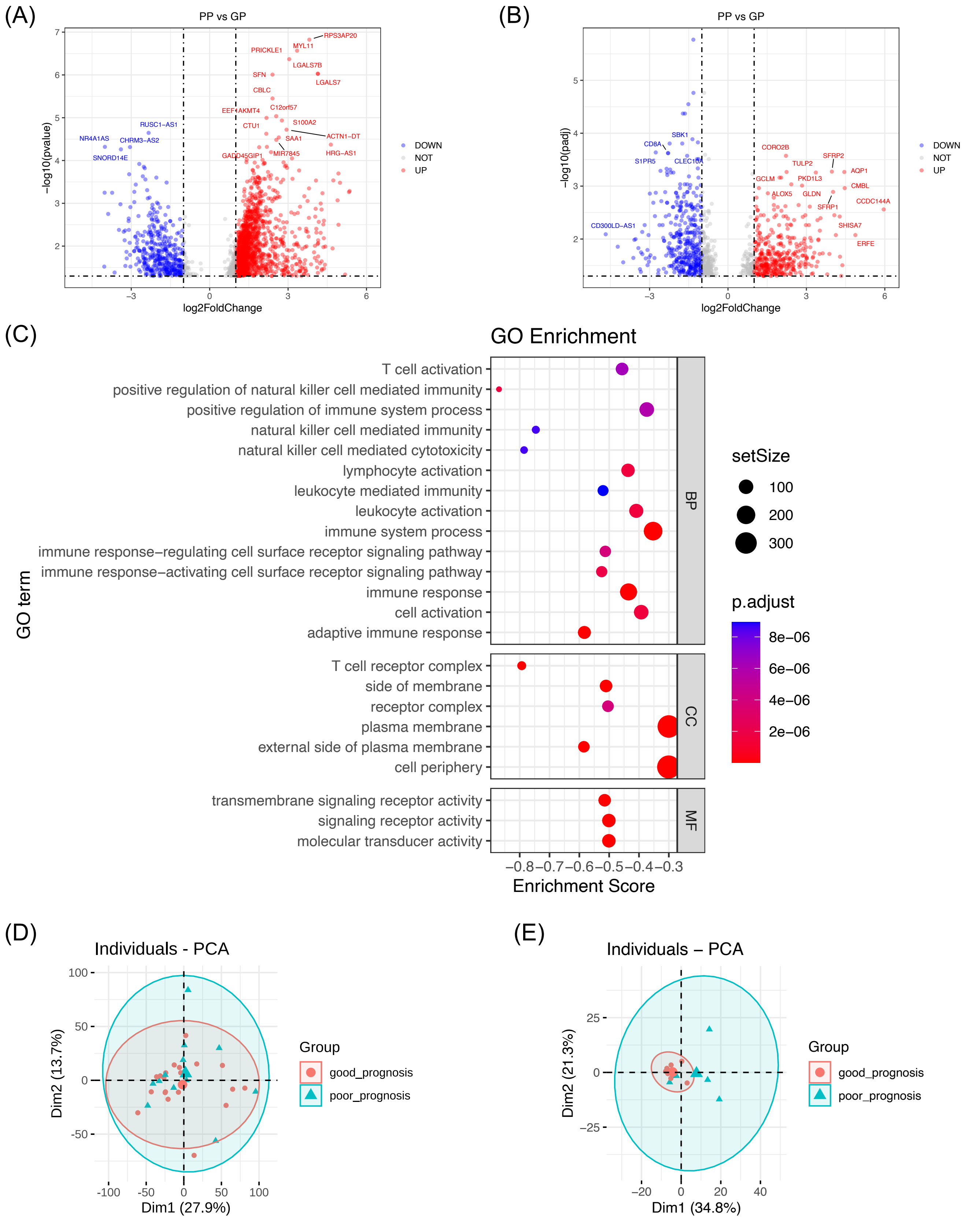

Figure S4 The immune landscape of asLRTI with different outcomes.
(A) Volcano plot of DEGs by RNAseq on BALF sediment in PP compared to GP; (B) Volcano plot of DEGs by RNAseq on PBMCs in PP compared to GP; (C) Enriched pathways in GO database were conducted on upregulated DEGs from RNAseq of PBMCs; (D) PCA plot of Proteome of BALF; (E) PCA plot of Proteome of serum.
p value was calculated by T-test.
p <0.05 was defined as significant.
Abbreviations: PP, poor prognosis; GP, good prognosis.

Figure S5

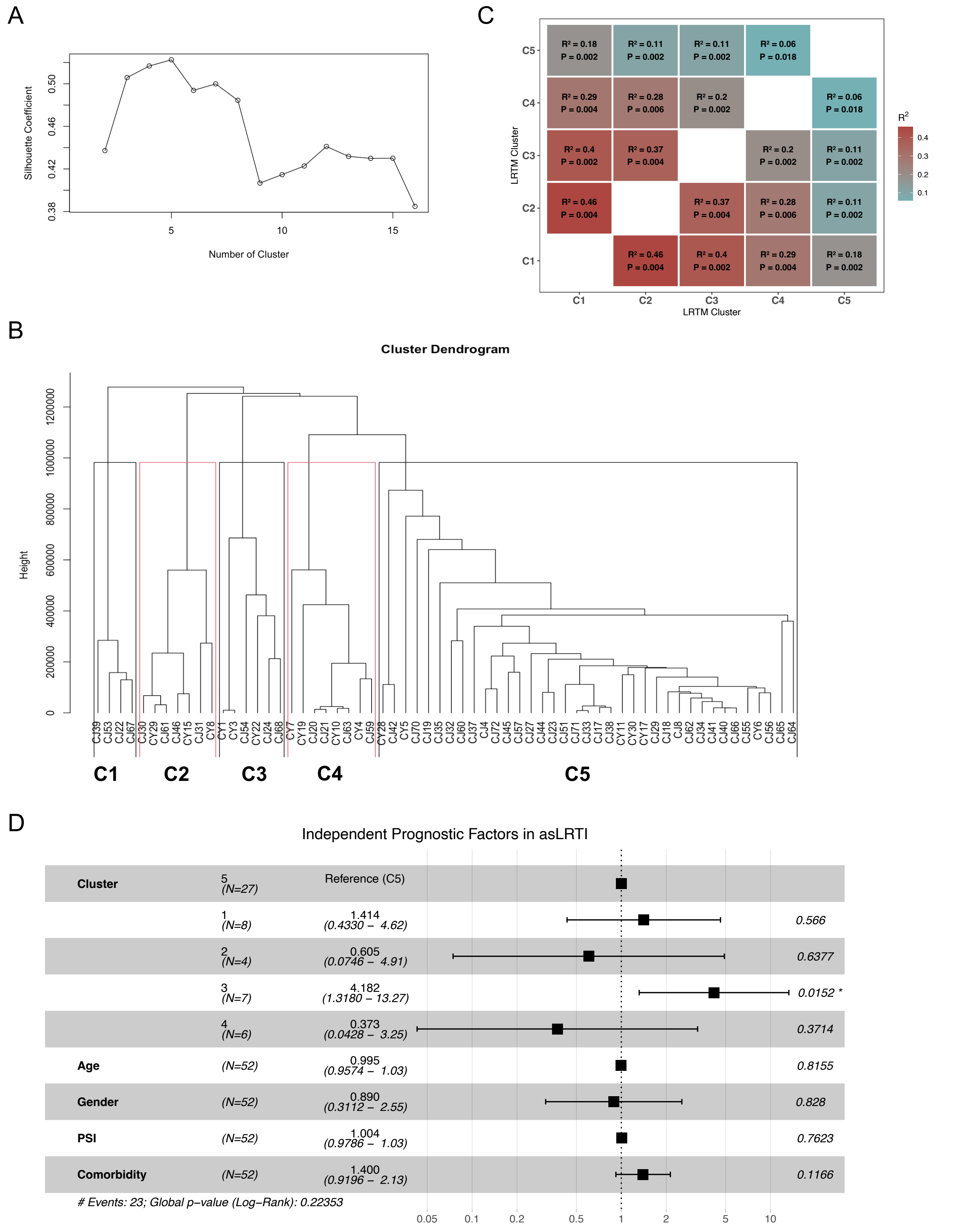

Figure S5 Effect evaluation for the cluster of LRTM in asLRTI.

(A) Plot of silhouette coefficient; (B) Cluster tree diagram; (C) Pairwise PERMANOVA analysis of microbial community structure across five LRTM clusters.Tile color represents R-squared (R^2) values from pairwise comparisons (red indicates higher R^2, representing greater community dissimilarity). Each tile shows R^2 and adjusted P-value (Benjamini–Hochberg correction).(D) Multivariate Cox regression forest plot for prognostic factors in patients with asLRTI. LRTM Cluster 5 was used as the reference group. Adjusted factors included age, gender, comorbidities, and pneumonia severity score. HR, hazard ratio; CI, confidence interval.

Figure S6

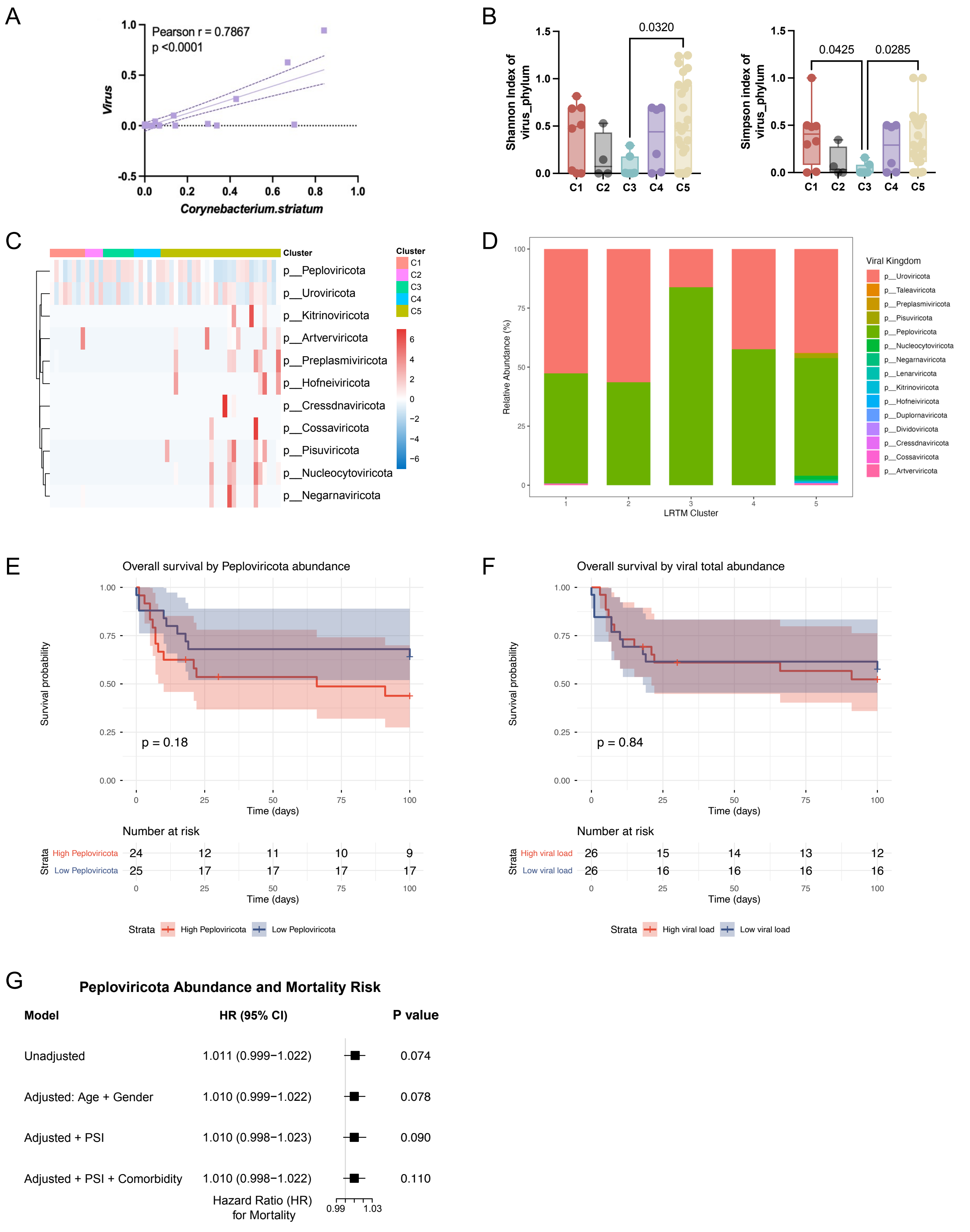

Fig S6 *Virus* profiles, correlations with *Corynebacterium striatum*, and prognostic associations.
(A) Pearson correlation between total *Virus* relative abundance and *Corynebacterium striatum*; (B) α-diversity (Shannon/Simpson indices) for *Virus* across LRTM clusters (Kruskal-Wallis test); (C) Heatmap of viral phylum abundance (Z-score normalization on phylum); (D) Stacked bar plot of average viral phylum composition per cluster; (E-F) Kaplan-Meier survival curves for high vs low (E) *Peploviricota* and (F) total *Virus* relative abundance; (G) Forest plot showing hazard ratios for mortality across sequential Cox models for *Peploviricota* abundance.

Supplemental TableS1 Baseline demographic and distribution of patients conducted multi-omics

		**asLRTIs**	**Controls**	**p value**
**BALF_mNGS**	No.	52	10	
	Gender, Male (n, %)	40 (76.9)	8 (80.0)	0.8416‡
	Age, Years (median, IQR)	73 (64, 79)	66 (58, 74)	0.2488#
**BALF_ RNAseq**	No.	41	16	
	Gender, Male (n, %)	34 (82.9)	12 (75.0)	0.7581‡
	Age, Years (median, IQR)	73 (67, 81)	65 (62, 69)	0.0571#
**BALF_DIA**	No.	33	7	
	Gender, Male (n, %)	27 (81.8)	6 (85.7)	0.7633‡
	Age, Years (median, IQR)	72 (67, 79)	62 (58, 74)	0.3261#
**BALF_ Metabolomics**	No.	18	4	
	Gender, Male (n, %)	13 (86.7)	2 (50.0)	0.5646§
	Age, Years (median, IQR)	72 (61, 78)	60 (52, 67)	0.1429#
**PBMC_RNAseq**	No.	15	10	
	Gender, Male (n, %)	11 (73.3)	7 (70.0)	>0.9999§
	Age, Years (median, IQR)	73 (68, 79)	69 (61, 74)	0.3107#
**Serum_DIA**	No.	14	5	
	Gender, Male (n, %)	11 (78.6)	4 (80.0)	>0.9999§
	Age, Years (median, IQR)	70 (55, 78)	64 (54, 73)	0.8734#

Abbreviations: BALF, bronchoalveolar lavage fluid; mNGS, Metagenomic next-generation sequencing; IQR, interquartile range; DIA, Data-Independent Acquisition Proteomics; SCFAs, Short-Chain Fatty Acids.

p values were calculated by T test (#), Yates' continuity corrected chi-square test (‡) and Fisher's exact test (§). $p < 0.05$ is considered as significant.

Supplemental Table S2 Clinical characteristics of patients with different prognosis

	Good prognosis	Poor prognosis	p value*
**No.**	29	23	
**Gender (Male, %)**	23 (79.3)	17 (73.9)	0.6464
**Age**	73 (64, 79)	72 (63, 80)	0.8585
**PSI**	108 (102, 116)	117 (102, 126)	0.2137
**Mechanical ventilation (%)**	24 (82.8)	21 (91.3)	0.3699
**Tracheotomy (%)**	15 (51.7)	13 (56.5)	0.7303
**Complications**			
Hypertension	12 (41.4)	13 (56.5)	0.2777
Diabetes	10 (34.5)	10 (43.5)	0.5078
Coronary heart disease	4 (13.8)	8 (34.8)	0.0744
Interstitial lung disease	3 (10.3)	3 (13.0)	0.7623
Hyperlipidemia	3 (10.3)	2 (8.7)	0.8412
Pulmonary tuberculosis	2 (6.9)	3 (13.0)	0.4552
COPD	1 (3.4)	1(4.3)	0.8670
Bronchial asthma	1 (3.4)	0 (0.0)	0.3685
Bronchiectasis	0 (0.0)	1 (4.3)	0.2569
**Smoke**	11 (37.9)	10 (43.5)	0.6855
**BALF culture positive**	16 (55.3)	18 (78.3)	0.0822
**Blood culture positive**	2 (6.9)	3 (13.0)	0.4552
**Laboratory tests**			
White blood cells (*10^9/L)	11.7 (8.2, 16.3)	11.1 (7.7, 15.7)	0.6217
Neutrophils (*10^9/L)	10.2 (6.1, 13.1)	10.1 (6.8, 14.6)	0.9709
Lymphocytes (*10^9/L)	1.3 (0.7, 2.5)	0.6 (0.5, 1.0)	**0.0023**
Hemoglobin (g/L)	101 (77, 130)	106 (87, 129)	0.5131
Platelet (*10^9/L)	227 (143, 286)	164 (103, 239)	0.0519
K^+ (mmol/L)	4.1 (3.6, 4.4)	4.2 (4.0, 4.7)	0.1469
Creatinine (mg/dL)	65.1 (39.1, 93.3)	83.1 (56.6, 156.3)	0.0672
BUN (mg/dL)	8.7 (5.5, 14.9)	11.6 (7.8, 20.3)	0.0893
CRP (mg/L)	76.5 (22.6, 168.5)	100.1 (70.33, 191.2)	0.2876
PCT (ng/ml)	0.3 (0.1, 0.9)	0.6 (0.1, 3.5)	0.3260

Abbreviation: asLRTI, acute severe lower respiratoty tract infection; PSI, Pneomonia severity index; COPD, Chronic Obstructive Pulmonary Disease; BUN, Serum urea nitrogen; CRP, C reactive protein; PCT, Procalcitonin.

* Continuous numerical data is shown as median (interquartile range) in this table, and categorical data are presented as n (%).The Mann Whitney test is used for comparison of continuous numerical data, while the Fisher's exact test is generally used for group comparison of categorical data. $p < 0.05$ is defined as statistically significant.

Supplemental table S3 Clinical characteristics of different lower respiratory tract microbiome characteristics in asLRTI

	Cluster_1	Cluster_2	Cluster_3	Cluster_4	Cluster_5	p value*
**No.**	8	4	7	6	27	
**Gender (Male, %)**	6 (75.0)	4 (100.0)	4 (57.1)	5 (83.3)	21 (77.8)	0.5731
**Age (Years)**	79 (66, 86)	67 (55, 83)	72 (53, 79)	74 (63, 87)	72 (66, 78)	0.7039
**PSI score**	114 (104, 125)	110 (106, 115)	102 (91, 119)	123 (100, 140)	108 (98, 121)	0.4503
**Tracheotomy (%)**	4 (50.0)	3 (75.0)	4 (57.1)	5 (83.3)	12 (44.4)	0.4256
**PICC (%)**	2 (25.0)	2 (50.0)	4 (57.1)	4 (66.7)	11 (40.7)	0.5438
**Complications (%)**						
**Hypertension**	3 (37.5)	2 (50.0)	1 (14.3)	4 (66.7)	15 (55.6)	0.2871
**Diabetes**	2 (25.0)	2 (50.0)	2 (28.6)	4 (66.7)	11 (40.7)	0.5464
**Coronary heart disease**	1 (12.5)	1 (25.0)	2 (28.6)	2 (33.3)	6 (22.2)	0.9101
**Bronchiectasis**	0 (0.0)	0 (0.0)	0 (0.0)	0 (0.0)	1 (3.7)	0.9182
**Hyperlipidemia**	1 (12.5)	0 (0.0)	1 (14.3)	1 (16.7)	1 (3.7)	0.6793
**Pulmonary tuberculosis**	2 (25.0)	0 (0.0)	0 (0.0)	0 (0.0)	3 (11.1)	0.3208
**Interstitial lung disease**	2 (25.0)	0 (0.0)	0 (0.0)	0 (0.0)	4 (14.8)	0.3529
**COPD**	1 (12.5)	0 (0.0)	0 (0.0)	0 (0.0)	1 (3.7)	0.6105
**Bronchial asthma**	0 (0.0)	0 (0.0)	0 (0.0)	0 (0.0)	1 (3.7)	0.9182
**Smoke (%)**	3 (37.5)	3 (75.0)	1 (14.3)	2 (33.3)	12 (44.4)	0.3660
**Outcomes-Good prognosis (%)**	4 (50.0)	3 (75.0)	1 (14.3)	5 (83.3)	16 (59.3)	0.1086
**Laboratory tests**						
**White blood cell (*10^9/L)**	8.3 (4.9, 14.9)	13.0 (8.3, 19.4)	12.2 (10.7, 19.8)	15.2 (11.0, 19.9)	11.6 (7.2, 13.8)	0.2994
**Neutrophils (*10^9/L)**	7.1 (3.9, 13.7)	10.6 (6.3, 16.2)	10.4 (7.5, 18.6)	11.2 (8.8, 16.5)	9.1 (6.1, 11.6)	0.4114
**Lymphocytes (*10^9/L)**	0.6 (0.5, 1.0)	1.9 (0.6, 3.3)	0.5 (0.4, 1.1)	1.8 (1.3, 2.8)	0.7 (0.6, 1.6)	0.0605

	Cluster_1	Cluster_2	Cluster_3	Cluster_4	Cluster_5	p value*
**CRP (mg/L)**	24.8 (2.5, 102.0)	83.8 (50.6, 148.2)	79.6 (41, 117.9)	66.9 (29.3, 116.4)	149.9 (48.3, 200.0)	0.0661
**PCT (ng/ml)**	0.7 (0.2, 11.4)	0.1 (0.1, 0.6)	0.3 (0.1, 3.2)	0.4 (0.2, 5.6)	0.3 (0.1, 1.9)	0.3646
**BALF culture positive (%)**	8 (100.0)	4 (100.0)	6 (85.7)	5 (83.3)	11 (40.7)	0.0034
**Blood culture positive (%)**	2 (25.0)	0 (0.0)	1 (14.3)	1 (16.7)	1 (3.7)	0.3785

Abbreviation: LRTIs, lower respiratory tract infections; IQR, interquartile range; PICC, peripherally inserted central catheter; COPD, chronic obstructive pulmonary disease; CRP, C-reactive protein; PCT, procalcitonin; BALF, bronchoalveolar lavage fluid.

* Continuous numerical data is shown as median (interquartile range) in this table, and categorical data are presented as n (%). The p-value for comparing multiple groups was calculated using the Kruskal Wallis test, and $p<0.05$ was defined as statistically significant.
